# Song and genetic divergence within a subspecies of white-crowned sparrow (*Zonotrichia leucophrys nuttalli*)

**DOI:** 10.1371/journal.pone.0304348

**Published:** 2024-05-29

**Authors:** Amy Rongyan Luo, Sara Lipshutz, Jennifer Phillips, Robb T. Brumfield, Elizabeth Perrault Derryberry

**Affiliations:** 1 Department of Ecology and Evolutionary Biology, University of Tennessee, Knoxville, TN, United States of America; 2 Department of Biology, Duke University, Durham, NC, United States of America; 3 School of the Environment, Washington State University, Pullman, WA, United States of America; 4 Museum of Natural Science and Department of Biological Sciences, Louisiana State University, Baton Rouge, LA, United States of America; McGill University, CANADA

## Abstract

Animal culture evolves alongside genomes, and the two modes of inheritance—culture and genes—interact in myriad ways. For example, stable geographic variation in culture can act as a reproductive barrier, thereby facilitating genetic divergence between “cultural populations.” White-crowned sparrows (*Zonotrichia leucophrys*) are a well-established model species for bird song learning and cultural evolution, as they have distinct, geographically discrete, and culturally transmitted song types (i.e., song dialects). In this study, we tested the hypothesis that divergence between culturally transmitted songs drives genetic divergence within Nuttall’s white-crowned sparrows (*Z*. *l*. *nuttalli*). In accordance with sexual selection theory, we hypothesized that cultural divergence between mating signals both preceded and generated genetic divergence. We characterized the population structure and song variation in the subspecies and found two genetically differentiated populations whose boundary coincides with a major song boundary at Monterey Bay, California. We then conducted a song playback experiment that demonstrated males discriminate between songs based on their degree of divergence from their local dialect. These results support the idea that discrimination against non-local songs is driving genetic divergence between the northern and southern populations. Altogether, this study provides evidence that culturally transmitted bird songs can act as the foundation for speciation by sexual selection.

## Introduction

Divergence in sexual signals can act as a reproductive barrier, and it has been theorized as a basis of speciation by sexual selection [1–5]. While speciation by sexual selection has been gaining traction as an idea, direct empirical evidence is not always clear [6]. In support of this hypothesis, behavioral discrimination and mate choice based on divergent sexual signals has been repeatedly demonstrated across a wide variety of taxa [4, 7]. Such discrimination and the potential for assortative mating have also been demonstrated for culturally transmitted traits (i.e., socially learned and passed through generations) [8]. Geographic concordance between population structure and sexual signal divergence is also used as evidence that discrimination is producing speciation by sexual selection. However, such evidence becomes less clear as the taxonomic resolution increases. It is possible that sexual trait divergence can only reinforce existing reproductive isolation; if so, one would expect to see areas of genetic divergence with or without sexual trait divergence [6]. Alternatively, weak population structure—which is predicted in early speciation by sexual selection—is difficult to detect without large sample sizes [9, 10].

Across a wide range of taxa, individuals discriminate between divergent sexual signals [4]. Preference for a sexual trait can be genetically linked to the trait itself [11, 12], but in other cases, preferences are learned through processes such as parental imprinting or social learning [13, 14]. For example, strawberry poison frogs (*Oophaga pumilio*) imprint on and prefer their mothers’ coloration [15], and female fruit flies (*Drosophila melanogaster*) use social learning to inform their preferences for male coloration [16]. In species that learn through social conformity, these preferences can become cultural traditions that are transmitted through generations within a population [16]. Thus, learned divergent sexual trait preferences can promote behavioral isolation between closely related taxa, and even intraspecific populations [5].

Such behavioral isolation can drive geographic concordance between populations and sexual signals. For instance, neighboring populations of the Amazonian frog *Physalaemus petersi* that have different male mating call types have lower gene flow, compared to neighboring populations with the same call type, which likely is a result of female preference for the local call type [17]. If genetic population structure is not detected, speciation by sexual selection is often ruled out [18–21], but population structure during early speciation by sexual selection can be weak and difficult to detect, while sexual trait variation is apparent [9, 10]. Further, if sexual trait boundaries are leading to genetic breaks (i.e., a discontinuity in a genetic area, or the boundary at which two genetic populations meet), they would be expected to precede them, as well. Genetic population boundaries—when present—would align with the underlying sexual trait boundaries.

Song is a sexual signal commonly theorized to promote speciation in birds [22]. Playback experiments have found that birds discriminate between conspecific and heterospecific songs [23–25], with males being the least aggressive toward highly divergent heterospecific songs [26, 27]. Within some species, individuals respond more strongly to local conspecific song types, compared to non-local conspecific songs [28–33]. However, behavioral responses to song divergence may vary. In some cases, the stronger response to the local dialect only holds for one sex, song type, or aggressive behavior [33, 34]. Further, salient aggressive signals may evoke less aggressive responses (e.g., intruder males will leave a territory in response to aggressive songs) [35]. Playback studies traditionally test for discrimination between categories of song. But there is evidence that, in some taxa, the strength of behavioral discrimination depends on continuous levels of acoustic dissimilarity [26, 27]. In such cases, one would expect the strength of behavioral responses to depend on the extent of acoustic dissimilarity to the local or conspecific song.

Other studies have recovered geographic concordance between intraspecific song variation and population structure. For example, greenish warblers (*Phylloscopus trochiloides*) are a ring species complex around the Tibetan plateau in which two distinct types meet in Siberia; the Siberian types differ strongly in their songs and rarely interbreed, so culturally-inherited song divergence appears to be acting as a prezygotic reproductive barrier [36]. But other empirical studies do not find evidence of intraspecific population structure aligned with song divergence [20, 37]. As signatures of genetic divergence become less perceptible within species, it becomes difficult to elucidate when song divergence can facilitate genetic divergence.

White-crowned sparrows (*Zonotrichia leucophrys*) have long been a model species for cultural evolution, or population-level changes in socially learned behaviors over generations [38]. The two coastal subspecies have well-described, distinct, and geographically discrete song types, generally referred to as dialects [39–43]. Juvenile white-crowned sparrow males learn songs from nearby adult tutors and are more likely to adopt common dialects [44]. While juveniles can learn multiple songs, adult males typically choose one song type to sing throughout adulthood, preferring to retain the song most similar to their neighbors [45]. This conformity bias leads to the formation of dialect regions, or discrete geographic areas in which most males sing highly similar songs. White-crowned sparrow dialects are categorized using their trill notes, which are highly similar between most males in a dialect area, but distinct between dialects. White-crowned sparrow dialects can remain stable and recognizable for decades, in spite of minor changes in song traits [46–48].

Evidence for speciation by sexual selection is mixed in white-crowned sparrows, despite the presence of discrete and spatiotemporally stable song dialects [49]. Between subspecies, Lipshutz et al. [29] found that song is a barrier to gene flow between Puget Sound (*Z*. *l*. *pugetensis*) and Nuttall’s (Z. *l*. *nuttalli*) white-crowned sparrows along the West Coast. While the barrier is porous, song divergence appears to be slowing admixture during secondary contact. However, these two subspecies also differ in song syntax and migratory behavior (*Z*. *l*. *pugetensis* is migratory, while *Z*. *l*. *nuttalli* is sedentary), indicating that song divergence may not be the only reproductive barrier between them. Within subspecies, there is support both for female preference for local dialects [30, 50] and concordance between population structure and dialect regions [21]. Other studies have found no relationship between song dialects and population structure [20, 51]. This may be due to dispersal across dialects or could be a sign that the populations are too early in the process of speciation by sexual selection to detect population structure from neutral markers [10].

In this study, we test the hypothesis that song divergence is restricting gene flow through assortative mating within continuously distributed populations of *Z*. *l*. *nuttalli*. Assortative mating can arise directly via female preferences for the local song dialect or indirectly via reduction in a male’s ability to hold a territory with a non-local song, thus limiting his ability to attract a mate [52]. *Z*. *l*. *nuttalli* are sedentary with finely structured cultural populations (i.e., each song dialect is sung over a small area). The more limited dispersal and exposure to other song dialects in *Z*. *l*. *nuttalli* make them a better candidate for speciation by sexual selection, compared to the other four *Z*. *leucophrys* subspecies, which are all migratory [53, 54]. Even low levels of dispersal between populations can prevent genetic divergence, so the relatively low dispersal of *Z*. *l*. *nuttalli* is more conducive to genetic drift between cultural populations [55, 56].

We first examined geographic concordance between population structure and song variation. If song divergence is facilitating genetic divergence, songs would diverge first, and the resulting population structure would align with the underlying song variation. In the early stages of reproductive isolation, this may appear as multiple song boundaries but little to no population structure. Therefore, we predicted the presence of more song boundaries than genetic boundaries, but that existing genetic boundaries would align with song boundaries.

We then experimentally evaluated behavioral responses to song divergence. Using a song playback experiment, we tested whether the strength of behavioral discrimination is due to continuous song divergence from the local dialect. Discrimination studies generally use treatments in which categories of foreign song can represent species or population identity [24, 25, 29], levels of acoustic dissimilarity to the focal males’ song [26, 27], or some combination of the two [27, 29]. To account for the potentially confounding effects of the singer’s genetic population, we included two non-local treatments: non-local song from the same genetic population and non-local song from another genetic population. The two non-local treatments are similar in their acoustic dissimilarity to the local song but differ in the singer’s genetic identity relative to the focal male. While our primary prediction is that the strength of behavioral discrimination depends on acoustic dissimilarity, the comparison of these treatments can test whether focal males are discriminating based on the singer’s genetic identity.

Male territorial behaviors are easier to assay in the field than females, and male aggression is positively correlated with female preference in white-crowned sparrows [15, 57]. However, in a territorial context, male and female Nuttall’s white-crowned sparrows respond differently to local and non-local dialects. While both sexes responded weakly to *Z*. *l*. *pugetensis* song and a distant dialect, females respond more strongly to the adjacent dialect across all aggressive behaviors, while males sang more in response to the local dialect and trilled and fluttered more in response to the adjacent dialect [34]. Additionally, male aggression itself is a measure of signal efficacy, as the facilitation of male-male competition is an important function of song [57]. A signal stimulus that elicits a stronger aggressive response is typically interpreted as reflecting greater stimulus salience [57, 58].

## Materials and methods

### Genetic sampling and sequencing

**Fig 1:**
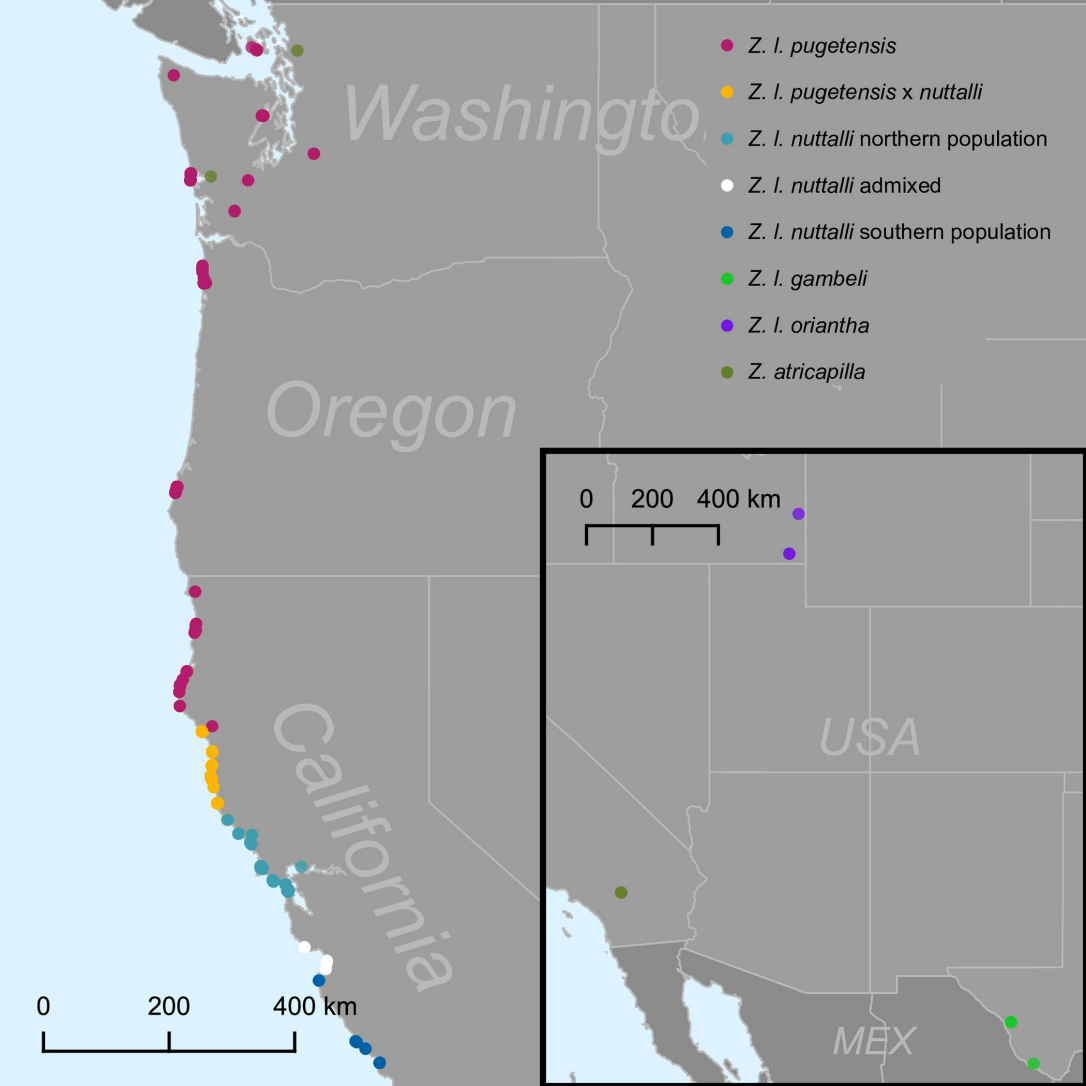
Map of all genetic samples before data filtering. Classification of *Z*. *l*. *nuttalli*, *Z*. *l*. *pugetensis*, and their hybrids is based on DAPC analysis of SNP data (see Population Structure methods below).

Genetic samples of *Z*. *l*. *nuttalli*, *Z*. *l*. *pugetensis*, and *Z*. *l*. *nuttalli* x *pugetensis* hybrids from 22 locations used in Lipshutz et al. (2017) were reanalyzed for this paper, along with additional samples collected from 11 locations in the hybrid zone, three additional locations south of the original sampling, and 12 individuals from three outgroups (Gambel’s white-crowned sparrows (*Z*. *l*. *gambeli*), mountain white-crowned sparrows (*Z*. *l*. *oriantha*), and golden-crowned sparrows (*Zonotrichia atricapilla*)). The entire dataset was used to delimit the full breeding range of *Z*. *l*. *nuttalli*, since the subspecies is admixing with *Z*. *l*. *pugetensis*; we defined the breeding range as the localities in which our discriminant analysis of principal components (DAPC) assigned multiple individuals to the *Z*. *l*. *nutalli* cluster. In total, we used 285 samples collected along the West Coast (Fig 1). 215 blood samples were collected during banding in 2004, 2005, and 2014. Dispersal is limited in the subspecies so there should be little, if any, change in population structure over the 10-year sampling period [55, 56]. In addition, 63 tissue samples were collected from vouchered specimens housed at the LSU Museum of Natural Science. Seven Genotype by Sequencing (GBS) samples were extracted from tissue loans from the University of California Berkeley Museum of Vertebrate Zoology (3 samples) and University of Washington Burke Museum of Natural History and Culture (4 samples).

Genetic samples were sequenced using GBS at the Institute of Genomic Diversity at Cornell University in Ithaca, NY, USA. Libraries were prepared following Elshire et al. [59], using the restriction enzyme PstI (CTGCAG) and a unique barcode for each sample on the plate. The first two GBS plates were analyzed in Lipshutz et al. [29], and a third plate of samples was extracted and sequenced following the same methods at the same facility. All three plates were processed and analyzed together in this study. We demultiplexed the GBS reads (i.e., sorted sequence reads into a separate file for each sample) for all three plates using the *process_radtags* function in STACKS [60], dropped samples with less than 500,000 reads, and called SNPs using the STACKS *ref_map* pipeline and a white-crowned sparrow draft genome as the reference. We filtered out SNPs with less than five percent minor allele frequency and more than 80 percent missing data using vcftools [61]. After data filtering, 263 individuals (251 focal samples and all 12 outgroup samples) and 26977 SNPs were retained in the dataset.

### Population structure

We first calculated pairwise Fst between sampling localities using the R package hierfstat [62]. The focal subspecies of this study, *Z*. *l*. *nuttalli*, hybridizes with another coastal subspecies, *Z*. *l*. *pugetensis*. We analyzed the same samples used by Lipshutz et al. [29] with the addition of more samples from the putative hybrid zone. We first ran fastSTRUCTURE [63] on all of the *Z*. *l*. *nuttalli*, *Z*. *l*. *pugetensis*, and *Z*. *l*. *pugetensis* x *nuttalli* samples. We ran the analysis using multiple numbers of clusters, k = 2 through k = 6, with 10 independent replicates for each k value, and estimated the optimal value of k using the *chooseK*.*py* script. We then averaged the results of the replicates of the optimal k value.

Clustering analyses like fastSTRUCTURE often recover two groups representing only the deepest divergence [64]. To estimate finer resolution population structure within the subspecies, we ran the analysis using only the *Z*. *l*. *nuttalli* samples identified by DAPC. We ran a DAPC using the adegenet R package [65] without the outgroup individuals. The *snapclust* function (maximum likelihood clustering) in adegenet found that k = 1 had the lowest BIC value, but the *find*.*clusters* function (K-means clustering) supported k = 2, with low assignment consistency for hybrids between replicates. This suggested support for two clusters but with unstable assignment for hybrids, so we ran a DAPC with k = 2 and hybridization. Parental groups and hybrids were assigned by snapclust, which we initialized with *a priori* subspecies assignments. Putative hybrids assigned to *Z*. *l*. *nuttalli* in the DAPC were included in subsequent analyses.

With the *Z*. *l*. *nuttalli* samples, we ran fastSTRUCTURE with k = 2 to k = 5 and used the *chooseK*.*py* script to estimate the optimal number of clusters. Using the optimal number of clusters identified by *chooseK*.*py*, we ran 100 fastSTRUCTURE replicates and averaged the 25 replicates that maximized the marginal likelihood. We repeated this process within each identified *Z*. *l*. *nuttalli* cluster until no further hierarchical population structure was found.

We located the geographic boundary between the two *Z*. *l*. *nuttalli* genetic populations by using ordinary kriging to spatially interpolate the fastSTRUCTURE output with the R package gstat [66]. We started by fitting a semivariogram on the admixture coefficients, or estimated amount of heritage from each ancestral population, of the samples. We then produced a regular grid of 10,000 points within California’s coastal scrub habitat [67] (bounded by the latitudes of the genetic samples), interpolated the ancestry coefficient at each grid point, and rasterized the point grid. Some predicted values were slightly above one or below zero, so they were truncated to one or zero to keep all values as proportions.

### Song sampling

**Fig 2:**
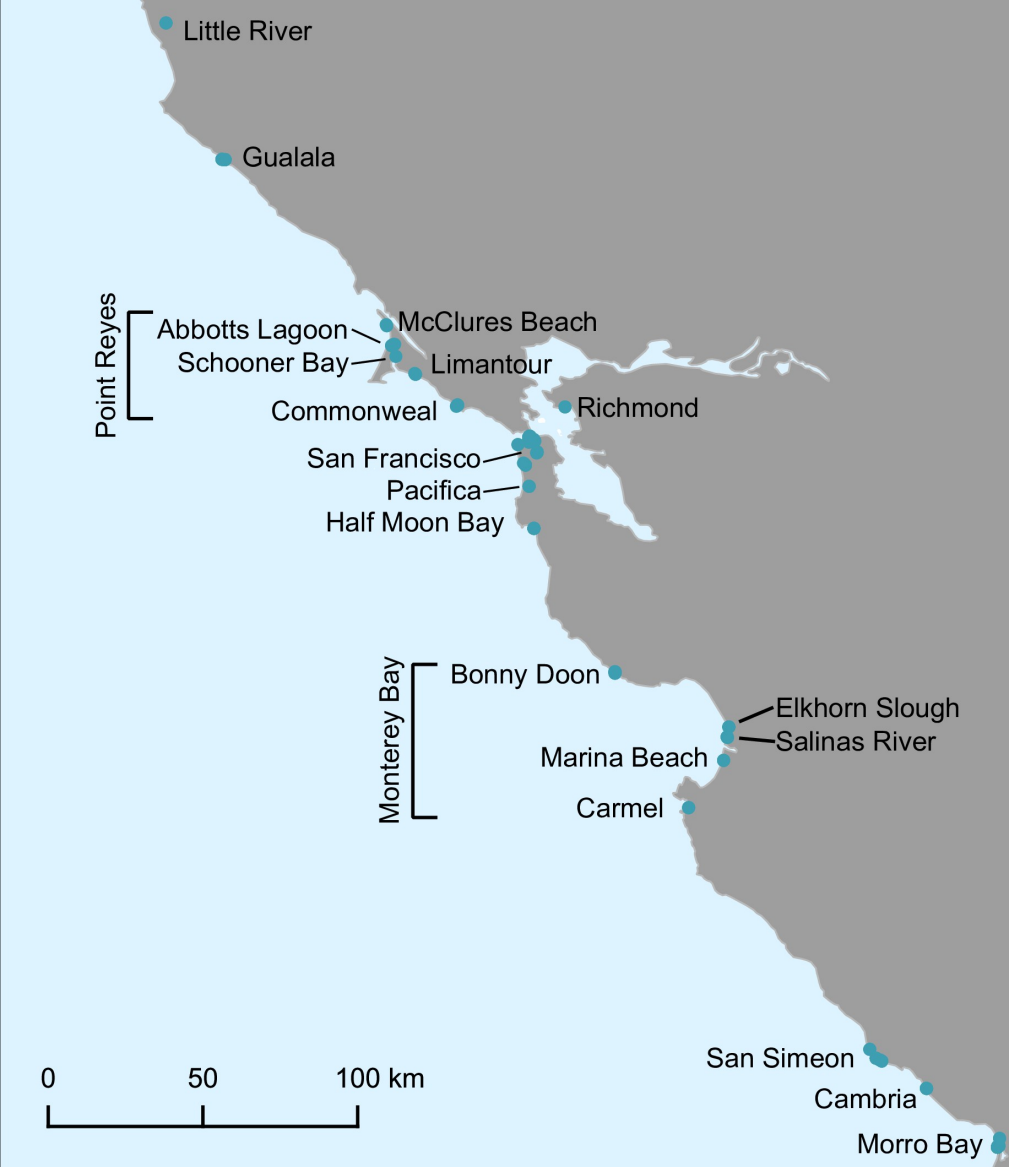
Map of song recording localities included in the song dissimilarity dataset. Dataset includes 175 songs (18 dialects) from 82 males of *Z*. *l*. *nuttalli*.

We used two sets of song recordings for different analyses. The first set (i.e., song dissimilarity dataset) included 175 songs from 82 *Z*. *l*. *nuttalli* males, representing 18 song dialects (i.e. 18 trill note types from different localities) recorded between 2010 and 2022 (Fig 2). Song dialects and genetic populations have both been stable for at least four decades, making the disparity in sampling times trivial [47, 68]. Songs were recorded during the breeding season within the *Z*. *l*. *nutalli* breeding range, as determined by the DAPC described earlier. Songs were pulled from the Derryberry Lab recording repository (153 songs from 72 males) and Xeno-Canto (22 songs from 10 males). We clipped songs from the Xeno-Canto recordings using Audacity 3.2.1. For all the songs, we removed low-frequency background noise in Audacity 3.2.1 using a high-pass filter between 1.5–2.5 kHz with a 48 dB/octave spectral rolloff.

**Fig 3:**
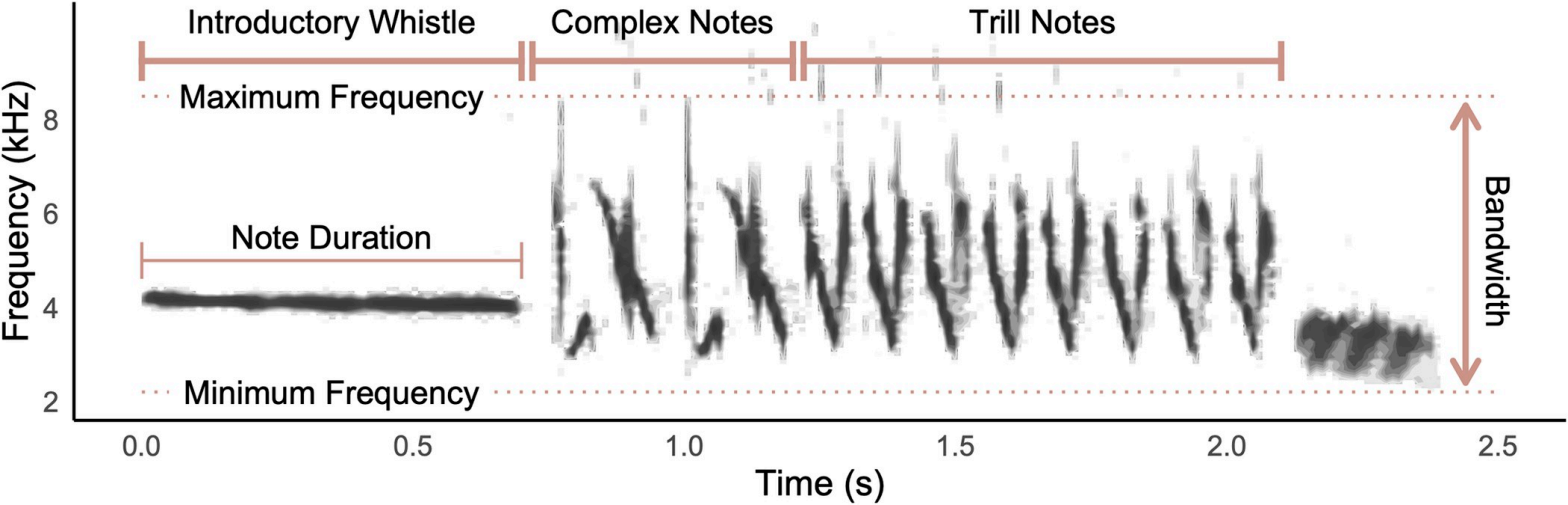
White-crowned sparrow song sections and song traits measured for PCA.

The second set of songs (i.e., song trait dataset) was comprised of 113 songs from 79 *Z*. *l*. *nuttalli* males; all the males in this dataset were also included in the GBS sequencing dataset. The songs were recorded between 2004 and 2014. We measured the durations of the whole song, introductory whistle, complex notes (mean duration), trill section, and individual trill notes (mean duration); bandwidth of the whole song, complex notes, and trill; maximum and minimum frequencies of the song, complex notes, and trill; trill rate; and dominant frequency of the introductory whistle (Fig 3). Measurements of song traits were taken in SIGNAL v5 [69], using spectrograms with a 256-point transform and frequency resolution of 97.7 Hz and 10.2 ms time resolution. The means of each measurement were calculated for each male.

### Song analyses

With the 175 songs in the song dissimilarity dataset (Fig 2), we calculated the dissimilarity between every pair of songs using the R package warbleR [70]. We first tracked the contours of the dominant frequency for each song; frequencies below 2 kHz and above 8 kHz were filtered out, and the fundamental frequency was sampled 200 times for each song, with a window length of 5000, 90% window overlap, 0% amplitude threshold for frequency detection, and frequency smoothing of 0.1 kHz. Using the frequency time series, we produced a pairwise dissimilarity matrix with a dynamic time warping (DTW) algorithm.

Given that song recordings from the repositories differ in their recording protocol, audio equipment, and audio quality, we wanted to ensure that this variation did not influence our analyses. Using pairwise dissimilarities from the DTW matrix, we determined whether the two source repositories produced artificial clusters in the songs. We ordinated the songs onto two axes using multidimensional scaling, then grouped the songs with k-means clustering and a maximum of two clusters. If the repositories are producing artificial clusters, songs from the same repository should cluster together.

We then found geographic boundaries between songs using the R package GeoOrigins [71] with a 95% confidence level for provenancing and Spearman’s correlation coefficient. Using sample coordinates and pairwise dissimilarity data, GeoOrigins estimates geographic areas in which sample values are highly correlated with neighboring samples. For each sample, boundary elements are defined as grid cells at which a minimum correlation coefficient threshold is crossed; grid cells that represent boundary elements for multiple samples are more intense geographic boundaries [71]. The placement and apparent strength of some boundaries may be partially an artifact of geographic sampling bias. For example, large gaps in sampling might produce a boundary in the gap, and “strong” boundaries might appear near heavily sampled areas. To assess the strength of the major boundaries, we compared the average song dissimilarity of each song to (1) songs from the same side of the boundary and (2) the opposite side of the boundary. We ran D’Agnostino tests for normality using the moments R package [72] and found that the datasets are all non-normal. Therefore, we ran one-tailed paired Wilcoxon signed-rank tests at each boundary to compare the same-side and across-boundary song dissimilarity.

GeoOrigins recovered a song boundary coincident with the genetic boundary (see Results below), so we performed a principal components analysis (PCA) to determine whether that boundary represents consistent song trait differences between the genetic populations. All measurements were scaled in the PCA, and only significant principal components (PCs), as determined using the Broken Stick method, were retained. For each retained PC, we ran D’Agnostino tests for normality and F-tests to compare variances for all genetic groups—the two genetic populations and the admixed group (i.e., from the area with genetic contributions from both populations). Each PC had non-normal groups or unequal variances, so we ran Kruskal-Wallis tests to compare the genetic groups [73]. We then ran Conover-Iman post hoc tests with Bonferroni corrections on PCs with significant Kruskal-Wallis tests.

### Playback experiment

We ran a playback experiment at six sites. Two sites each were located within the (1) northern *Z*. *l*. *nuttalli* population (Abbotts Lagoon and Commonweal, in Point Reyes National Seashore), (2) southern *Z*. *l*. *nuttalli* population (Morro Bay and San Simeon State Parks), and (3) admixture area between the populations (Moss Landing and Bonny Doon State Beaches) S1 Fig. Song stimuli were drawn from recordings produced between 2004 and 2020.

Focal males were presented with a sequence of three stimulus periods within a 24-minute trial. Each stimulus period represented one treatment: (1) a local song, (2) a non-local song from the same genetic population, and (3) a non-local song from the other *Z*. *l*. *nuttalli* population. All admixed males heard a local song and a non-local admixed song, but 10 males heard non-local songs from the northern population, and 7 heard non-local songs from the southern population. Treatment order was randomized for each male to compensate for order effects, and stimulus sequences were prepared in advance, so that we scored the behaviors while blind to the treatments. Neighbors were tested on different days so that they did not become habituated to the stimuli during tests on neighboring males. To avoid pseudo-replication [74], every male heard a unique set of stimuli, and every song was used only once.

We placed a speaker (black Ultimate Ears Wonderboom 3) in a male’s territory, near a perch on which we observed him singing, to broadcast the stimuli. Starting from the speaker, we rolled out two ropes flagged at 2m, 4m, 8m, and 16m; the ropes were laid out at roughly 90°-180° from one another, depending on the vegetation structure of the male’s territory. Song amplitude was normalized and broadcast at a level typical of free-living males (80–82 dB sound pressure level at 1m). The stimulus periods were each three minutes long, with one song played repeatedly at a natural rate of six songs per minute. Each stimulus period was separated by a six-minute “cooldown” period. Focal male behavior was observed during the three-minute playback periods and for three minutes after each playback period. We measured four response variables: mean distance from the speaker during the (1) playback period and the (2) post-playback period, (3) number of flights over the speaker during the playback period and (4) number of songs during the playback period. Distance from the speaker was estimated in meters every 10 seconds within distance intervals (0-2m, 2-4m, 4-8m, 8-16m, and >16m), using the flagged ropes as references for these intervals. The middle distance within the interval was used as the value (e.g., 1 m for the nearest interval). These mean distance for a period was calculated across all estimates within the period.

We ran a PCA on the four behavioral variables, all scaled, to extract an aggression metric. We used PC1 as our aggressive response variable, since higher values of PC1 indicate increased aggressive behavior (e.g., increased song rate, smaller distance to speaker). We then calculated behavioral discrimination between songs as the difference in aggression between the local and non-local treatments (discrimination = PC1_local_−PC1_foreign_), leaving two data points for each male: discrimination against each non-local song. For each male tested, we calculated song dissimilarity between the local stimulus and each foreign stimulus using a dynamic time warping algorithm, following the methods under the song analyses section. We then normalized song dissimilarity using z-scores, so the magnitude of variation would more closely match the Fst values. Two out of 64 focal males we tested were not present for all three trials. Specifically, they did not show up until after the first song stimulus was over. It is difficult to differentiate between a lack of motivation and a lack of perception in these cases, so they were removed from the statistical analysis.

If the treatments differed in both their acoustic dissimilarity and genetic identity (i.e., all the males from the other genetic population also had more dissimilar songs), the genetic divergence would confound the potential effect of song divergence on behavioral discrimination. Therefore, we aimed to decouple genetic population and acoustic dissimilarity in this experiment. We selected song stimuli to ensure that the non-local treatments contained similar levels of acoustic dissimilarity to the local song treatment. The treatments differed in genetic divergence from the focal male but not song divergence. Rather, similar levels acoustic dissimilarity from the local dialect were present within each treatment, which we confirmed with a t-test. To determine whether songs from the other genetic population were less salient signals, regardless of acoustic dissimilarity, we ran an ANOVA comparing the aggression metric (PC1) across the three treatments. Irrespective of whether males discriminate between categories of song, our focus is to understand how males respond to continuous variation in song that exists in both treatments.

We then tested whether acoustic dissimilarity or Fst could predict the aggression of focal males toward the song stimuli. Pairwise Fst values were calculated for each site based on the GBS data, not these experimental males. For 11 focal males, one of the treatments was missing an Fst value; the foreign song stimulus was drawn from a population without genetic data. Data points for those 11 males were initially removed from the analysis. We first ran a linear mixed model (LMM) to test whether song dissimilarity is correlated with Fst or geographic distance (reduced dataset: n_males_ = 51), using the R package lmerTest [75] with focal male and dialect comparison as random effects. For the dialect comparison, each factor was the combination of the focal male’s locality and the dialect of the song stimulus (e.g., focal males from Abbotts Lagoon that responded to a Morro Bay stimulus song, or focal males from Moss Landing that responded to a San Francisco stimulus song). We used dialect comparison as a random effect to account for non-independent genetic and song sampling, since both sample types were drawn from the same localities.

Since the two predictors are uncorrelated, we then ran an LMM with behavioral discrimination as the response variable, using only focal male as the random effect, as the song stimuli are unique to each trial (reduced dataset: n_males_ = 51). We compared the corrected Akaike Information Criterion (AICc) values of LMMs with different fixed effects—song dissimilarity, Fst, or both—using the *dredge* function in the MuMIn package [76], then ran the LMM with the best (i.e., lowest) AICc value. Because the best model included only song dissimilarity as a fixed effect, we included the males with the missing Fst values in the final model (n_males_ = 62).

### Ethics statement

All fieldwork was conducted on public lands, and no endangered or threatened species were tested. Genetic samples were collected under Duke University Institutional Animal Care and Use Committee (IACUC) Protocol A099-06-03 and Louisiana State University IACUC protocol 10–009. Fieldwork in 2004 and 2005 was conducted under Federal Fish and Wildlife Banding Permit 22712-G and Collecting Permit MB-813248, California Scientific Collecting Permit 801208–05, Oregon Scientific Taking Permit 050–04, and Washington State Scientific Collection Permit 04–110. Fieldwork in 2010 was conducted under Federal Fish and Wildlife Collecting Permit MB-679782-2 and California Scientific Collecting Permit 802010–02. Fieldwork in 2014 was conducted under the same Federal Fish and Wildlife Collecting Permit, California Scientific Collecting Permit SC-6799, and California Department of Parks and Recreation permit 22-820-004. Playbacks were conducted in 2022 under University of Tennessee IACUC Protocol 2792–1220, National Parks Service IACUC Protocol CA_GOGA.PORE_Derryberry_Sparrow_2021.A2, National Parks Service research permit PORE-2021-SCI-0013, California Scientific Collecting Permit S-202380004-20337-001, and California Department of Parks and Recreation permit 22-820-004.

## Results

### Population structure

**Fig 4:**
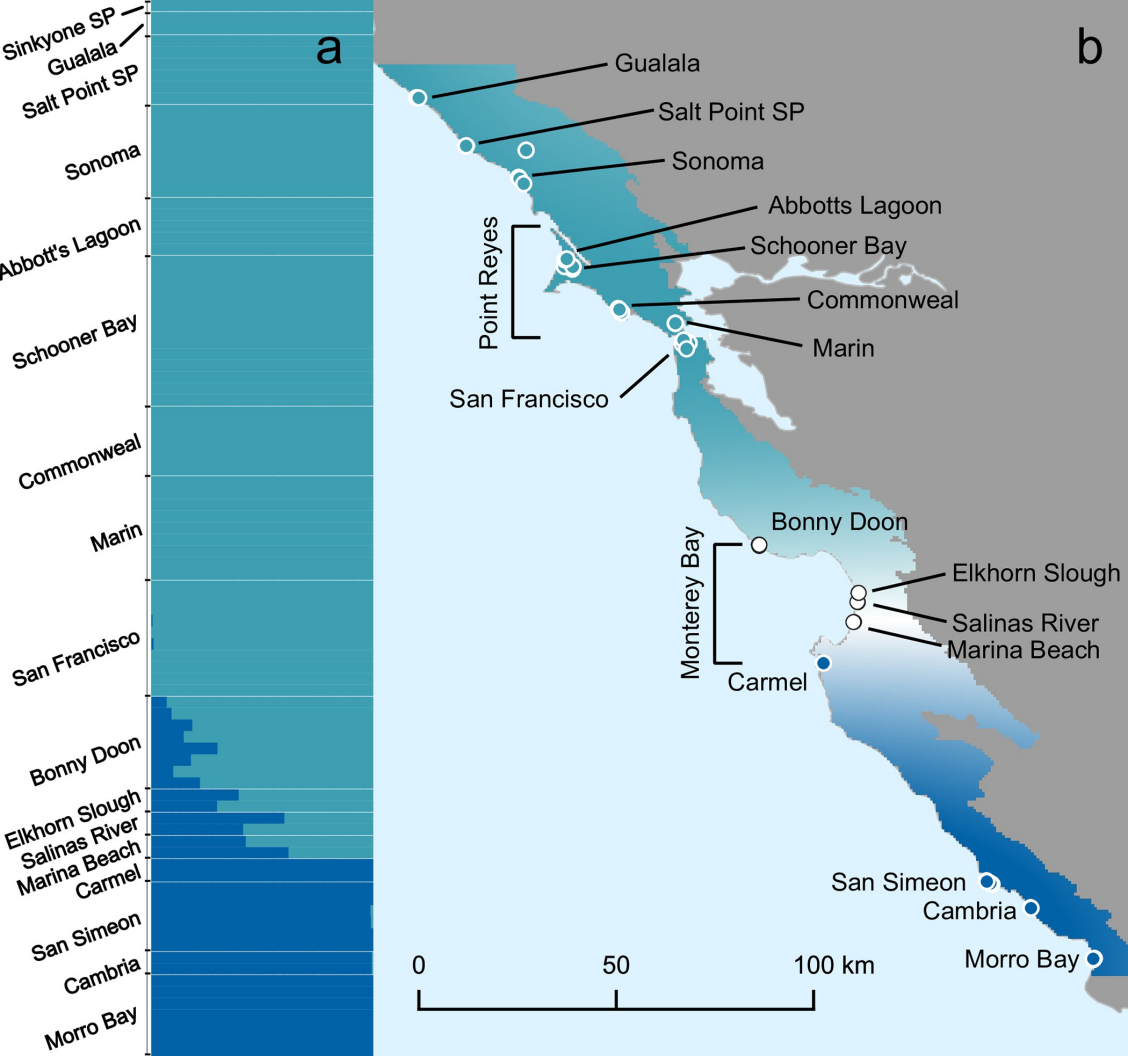
Population structure of *Z*. *l*. *nuttalli*. Light blue indicates the northern population and dark blue the southern population. (A) fastSTRUCTURE assignment probabilities, sorted north to south. (B) Interpolated assignment probabilities to northern and southern *Z*. *l*. *nuttalli* populations, with the white band at Monterey Bay representing admixture.

fastSTRUCTURE identified k = 2 as the optimal k value for *Z*. *l*. *pugetensis* and *Z*. *l*. *nuttalli* samples, and as in Lipshutz et al. [29], the two subspecies were found to be genetically distinct but admixing S2 Fig. The DAPC reassigned 17 putative hybrids to *Z*. *l*. *nuttalli* and 35 to *Z*. *l*. *pugetensis* S3 Fig. The individuals reassigned by the DAPC to the parental clusters were at the edges of hybrid zone, effectively narrowing the putative hybrid zone. fastSTRUCTURE recovered two *Z*. *l*. *nuttalli* clusters, northern and southern (Fig 4A). Further runs on subsets of *Z*. *l*. *nuttalli* samples found no additional population structure. Admixed individuals were found around Monterey Bay, from Bonny Doon to Marina Beach (Fig 4). Carmel, on the southern part of Monterey Bay, had relatively high pairwise Fst values across population comparisons S4 Fig.

### Song analyses

**Fig 5:**
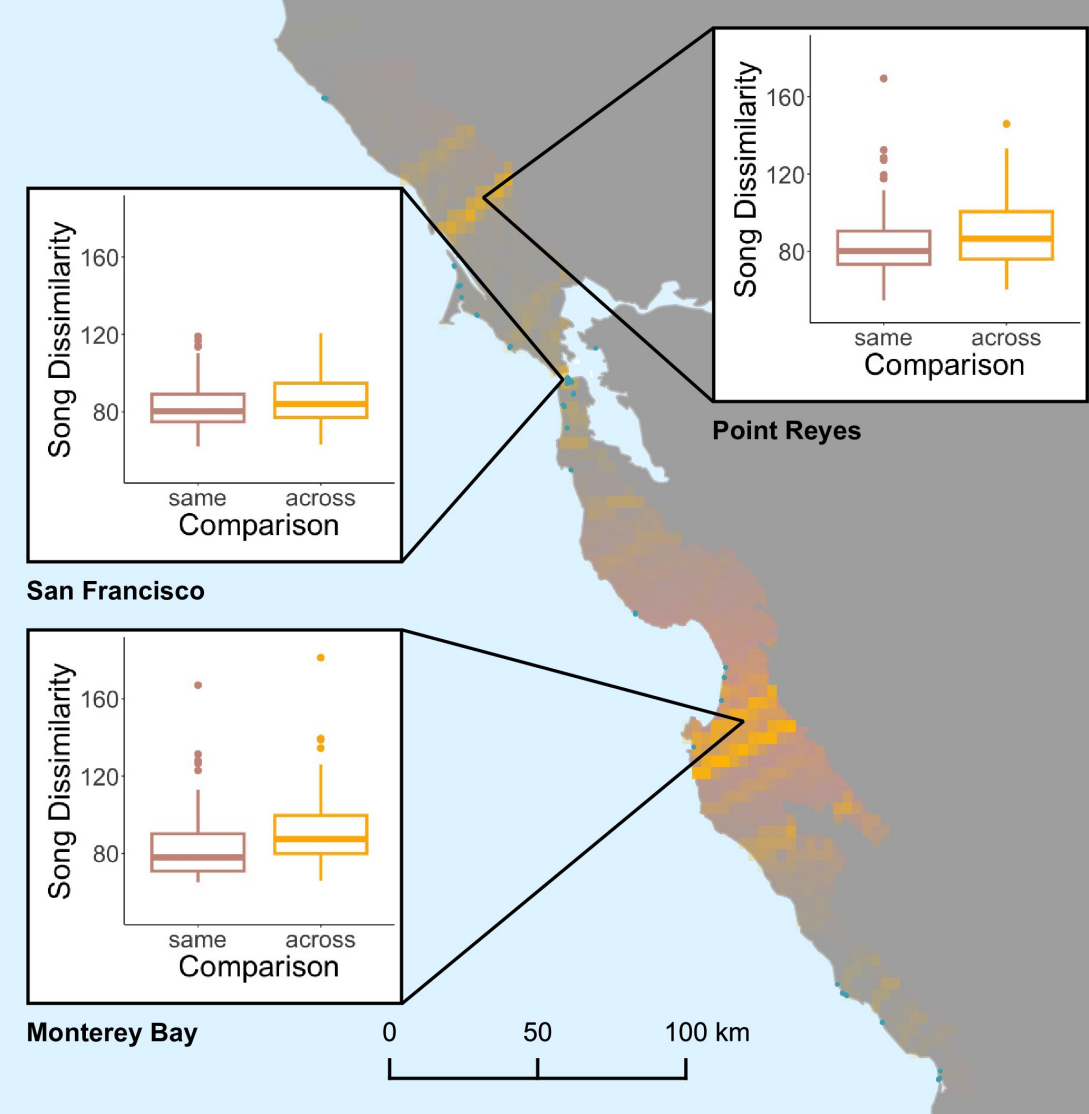
Song boundaries and acoustic dissimilarity comparisons at boundaries. Points are sampled songs, and yellow cells represent boundary elements, with darker yellow indicating more intense boundaries. Pink shading shows admixture at the genetic boundary, with the darkest pink indicating an assignment probability of 0.5. Insets show comparisons of mean across-boundary and same-side acoustic dissimilarity.

We recovered three major song boundaries at Point Reyes, San Francisco, and Monterey Bay using GeoOrigins (Fig 5). We found no evidence that variation in recording protocol or audio quality between repositories produced artificial clustering in the songs S7 Fig; these boundaries do not appear to be artifacts of sampling from multiple repositories. These boundaries roughly delineate four cultural populations, which is notably fewer than the 18 sampled song dialects. Using paired one-tailed Wilcoxon signed-rank tests at each boundary, we found significantly higher song dissimilarity to songs across the boundary, relative to dissimilarity to songs on the same side of the boundary (Monterey Bay, n = 143, effect size = 8.56, V = 8670, p < 0.001; San Francisco, n = 117, effect size = 3.15, V = 5719, p < 0.001; Point Reyes, n = 166, effect size = 6.90, V = 9673, p < 0.001; α = 0.05). In other words, songs from the same side of a boundary were, on average, significantly more similar to each other than to songs from the other side of the boundary. This comparison was the most stark at the Monterey Bay boundary (Fig 5).

**Fig 6:**
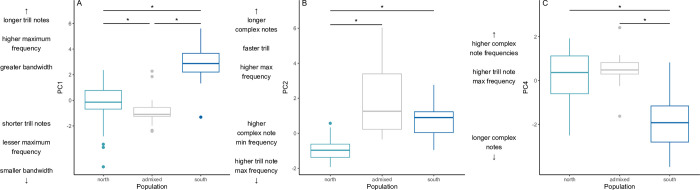
Song trait principal components by genetic population. (A) PC1, for which high values indicate higher song maximum frequencies and longer trill notes; (B) PC2, for which high values indicate higher minimum frequencies and longer complex notes; and (C) PC4, for which higher values correspond to higher minimum trill frequencies. Each PC is shown separated by genetic *Z*. *l*. *nuttalli* group. Asterisks indicate statistically significant differences between the groups.

The first four principal components of the song trait PCA were found to be statistically significant using the Broken Stick method. PC1 explained 21.43% of the variance, PC2 explained 20.78%, PC3 explained 13.70%, and PC4 explained 10.95% S5 Fig. PC1, PC2, and PC4 had significant differences between some genetic groups within *Z*. *l*. *nuttalli* (Kruskal-Wallis tests: *X*^2^ = 21.49, *X*^2^ = 56.1, and *X*^2^ = 14.0, respectively; df = 2, p < 0.001, α = 0.05 for all tests). The northern and southern populations were significantly different from each other along all three of these PCs (PC1: Z = -4.11, PC2: Z = -6.02, and PC4: Z = 3.73; p_adj_ < 0.001, α = 0.05 for all tests) (Fig 6). The admixed group was significantly different from both the northern and southern populations along PC1 (admix-north: Z = -2.51, p_adj_ = 0.0212, admix-south: Z = -5.37, p_adj_ < 0.001) (Fig 6A), only the northern population along PC2 (Z = 9.66, p_adj_ < 0.001) (Fig 6B), and only the southern population along PC4 (Z = 3.87, p_adj_ < 0.001) (Fig 6C).

PC1 was strongly and positively loaded with the bandwidth for the overall song and trill notes; maximum frequency of the overall song, complex notes, and trill notes; and trill note length. It is negatively loaded with trill rate. PC2 has strong positive loadings for complex note length, trill rate, and overall minimum frequency, and is negatively loaded with the minimum frequency of complex notes and maximum frequency of trill notes. PC4 has strong positive loadings for minimum and maximum complex note frequencies and trill minimum frequency; it is strongly and negatively loaded with complex note length S5 Fig. In short, higher values of PC1 are associated with longer and higher frequency notes with higher bandwidth, which are inversely related to trill rate; higher values of PC2 are associated with disparate traits, though largely minimum and maximum frequencies; and like PC1, higher values of PC4 are associated with higher frequencies and shorter notes.

### Playback experiment

We first ran a PCA to collapse the four behaviors into a single metric for aggression. We used PC1, which explains 53.11% of variance in the behavioral response, as the aggression score. Higher values of PC1 represent more songs and flights and proximity to the speaker S6 Fig. Therefore, a high PC1 is indicative of high aggression. Our ANOVA found no significant difference between responses to the three categorical stimulus treatments (F = 0.008, p = 0.992). The non-local song treatments overlapped in their degree of acoustic dissimilarity to the local song treatment (paired t-test: t = 0.080794, df = 61, p = 0.9359). Therefore, this analysis does not account for the continuous variation in song divergence that we hypothesize is driving male discrimination.

**Fig 7:**
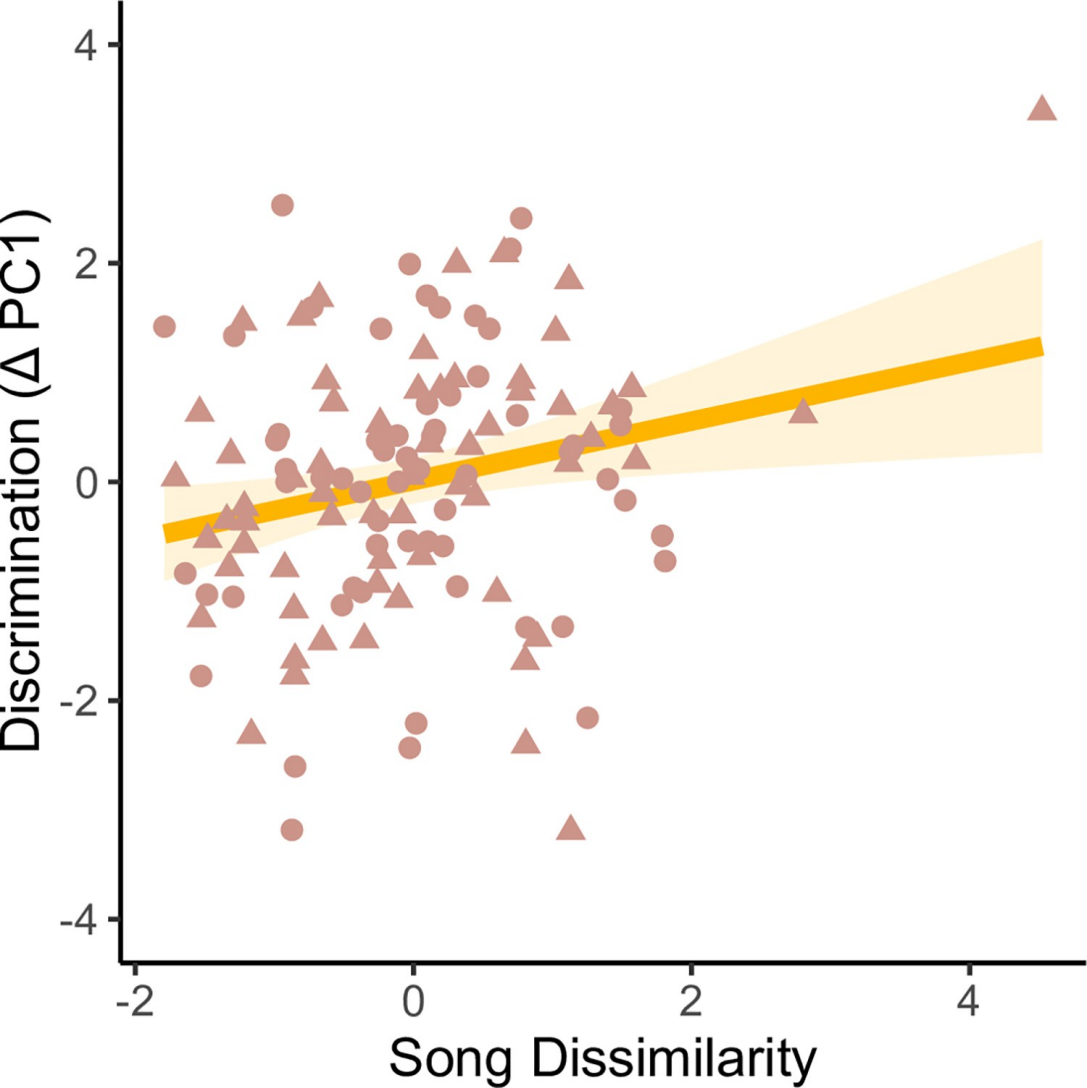
Correlation between behavioral discrimination and song dissimilarity. Each male is represented by two points: discrimination toward a non-local song (circles) from the same population and toward a song from the other population (triangles).

To assess the effects of continuous song dissimilarity—calculated using a dynamic time warping algorithm—on behavioral discrimination in males, we ran an LMM. In our full model, local males’ behavioral discrimination between local and foreign songs was predicted by Fst and song dissimilarity. When the models were competed against each other, the model with the lowest AICc retained only song dissimilarity as a fixed effect. The LMM with the reduced dataset (only focal males with associated Fst values, n_males_ = 51) recovered a positive but nonsignificant trend between discrimination and song dissimilarity (effect size = 0.182, se = 0.107, t = 1.71, p = 0.0907). When we added the males with missing pairwise Fst values back into this model, we found a significant positive relationship between discrimination and song dissimilarity (n_males_ = 62, effect size = 0.255, se = 0.0912, t = 2.79, p = 0.00625, α = 0.05) (Fig 7, S2 Appendix).

In the case that acoustic dissimilarity is a function of genetic or geographic distance, the effect of acoustic dissimilarity would be confounded by these variables. But based on an LMM, acoustic dissimilarity between songs was not correlated with either predictor (Fst: effect size = -0.560, se = -0.41, t = -1.37, p = 0.185; geographic distance: effect size = 1.32x10^-7^, se = 8.18x10^-8^, t = -1.65, p = 0.104). In other words, differences in song between populations is not explained by the genetic distance or the geographic distance between those populations. Therefore, the relationship between acoustic dissimilarity and behavioral discrimination appears to be independent of genetic and geographic distance.

## Discussion

Culturally-inherited mating signals mediate assortative mating across taxa [8, 29, 33, 77]. Conspecific songs are more salient signals for attracting females and holding territories (i.e., male competition), so interspecific song divergence can reduce gene flow across taxa [1, 2, 7, 22–24, 26, 27]. There is also evidence that sexual selection against divergent mating signals can produce reproductive isolation *between* subspecies [29, 78]. But the ability of song to reduce gene flow *within* subspecies or populations has remained unresolved. Here, we investigated whether song has the potential to reduce gene flow within the subspecies *Z*. *l*. *nuttalli*. Our results are consistent with early stages of speciation by sexual selection.

As predicted under sexual selection theory [10], white-crowned sparrows show more “cultural population structure” than genetic population structure, along with concordant cultural and genetic boundaries. We identified three major song boundaries in Point Reyes, San Francisco, and Monterey Bay. We also recovered one genetic boundary separating northern and southern genetic populations within *Z*. *l*. *nuttalli*. These boundaries are consistent with predictions that sexual trait divergence arises first in the speciation process, producing an initial disparity between cultural and genetic variation [79]. It is important to note that the different number of boundaries for song and genetic data could be a function of the different statistical tools used; however, it is unlikely that fastSTRUCTURE missed additional genetic boundaries given the low genetic differentiation among populations, except at Monterey Bay. The different number of boundaries could also be due, in part, to the genetic data collection method we used. For example, future genome resequencing could uncover geographically structured genetic variation we did not detect using GBS.

The genetic boundary is concordant with the song boundary at Monterey Bay, which is the most prominent song boundary in terms of acoustic dissimilarity. Lipshutz et al. [29] also found geographic concordance between song and genetic divergence in white-crowned sparrows, and that males discriminate between songs from their own subspecies and the other subspecies. Together, these studies suggest that song divergence can act as a reproductive barrier in white-crowned sparrows at two stages of genetic divergence: between the subspecies *Z*. *l*. *nuttalli* and *Z*. *l*. *pugetensis*, and between populations within *Z*. *l*. *nuttalli*. We posit that these two points of concordance indicate that culturally inherited song divergence can produce reproductive barriers through sexual selection, though we suggest further studies examining geographic concordance between genetic and song boundaries—especially those that focus on learned songs and multiple stages of speciation.

Given the presence of 18 discrete geographically structured song dialects in our dataset, as determined by visual inspection of spectrograms, it was unsurprising that GeoOrigins identified multiple song boundaries. The recovered boundaries do not separate all *Z*. *l*. *nuttalli* dialects but group some neighboring dialects together. Given the wide latitudinal range of the songs, the sampling density or resolution of the analysis may not have been fine enough to separate all the dialects. Alternatively, these larger groupings may represent “superdialects,” similar to the Puget Sound subspecies [40]. Unlike *Z*. *l*. *pugetensis*, the dialects in *Z*. *l*. *nuttalli* generally do not vary syntactically. Whether the grouping is due to low resolution in the analysis or the presence of inter-dialect similarity, these results are consistent with early speciation by sexual selection since they establish more cultural populations than genetic populations.

For our playback experiment, we interpreted a weaker male response as an indicator of lower signal salience of the stimulus (i.e., songs that elicited less aggression are less salient in the context of territorial defense). Under this interpretation, our results suggest that songs that are more divergent from those of the focal male are less salient; male responses to foreign stimuli were lower relative to their responses to the local dialect. Males singing such songs should be less able to acquire and maintain a territory in the same song neighborhood, which should reduce gene flow between areas with divergent songs.

However, it is possible to interpret the strength of response in the opposite direction. A weaker male response would instead indicate higher signal salience of the stimulus (i.e., songs that elicited less aggression are more salient signals in the context of territorial defense, for instance in cases of de-escalation [35]). With this interpretation, our playback results suggest that songs that are more divergent from the focal male’s song are in fact more salient, so males singing highly divergent songs could successfully compete for territories against males singing the local dialect. If this is the case, song divergence would not restrict gene flow, and we would not expect to find concordant genetic and song boundaries. Regardless of whether highly salient signals elicit stronger or weaker male responses, the difference in responses between stimuli suggests differing levels of salience [35]

The genetic differentiation at Monterey Bay might be maintained by acoustic dissimilarity through sexual selection and assortative mating. Together, the playback results and the genetic and song boundary overlap suggest songs that are highly dissimilar from the local dialect may be less salient mating signals. Studies focusing on the behavioral discrimination between songs generally compare categorical treatments, but these treatments either represent different genetic identities, levels of acoustic dissimilarity, or both [26, 27]. Here, the two non-local song treatments represented different genetic identities but similar ranges of acoustic dissimilarity from the local song dialect. The results of our playback experiment demonstrate that males responded similarly to the non-local treatments, suggesting that they did not discriminate between songs based on the genetic identity of the singer. Instead, males discriminated more strongly as the non-local song became more acoustically dissimilar from the local song. While the recovered effect of acoustic dissimilarity is weak, these are contiguous populations. These results suggest the early signatures of behavioral discrimination between song dialects based on acoustic divergence, even between continuous populations of a single subspecies.

Songs that elicit a weaker response from males—in this case, songs that are more acoustically dissimilar to the local dialect—may be less salient territorial defense signals, thereby limiting mating opportunity [52]. Moreover, female preference is positively correlated with male aggression in white-crowned sparrows [57]. Therefore, females may base their mating preferences on acoustic dissimilarity, further driving assortative mating based on acoustic dissimilarity.

We posit that the greater song divergence at Monterey Bay has produced sufficiently strong sexual selection to drive concordant genetic divergence. The song boundary at Monterey Bay is stronger than the others; it has the greatest acoustic dissimilarity to songs from the other side of the song boundary, relative to songs from the same side of the boundary. Why we did not observe genetic differentiation across the Point Reyes and San Francisco remains unclear, though. The other two song boundaries may be younger or less spatiotemporally stable than the Monterey Bay song boundary. We know the genetic break between northern and southern populations has been present at Monterey Bay for at least 40 years [68], which is approximately 40 generations for this species. Unfortunately, longitudinal data that would allow us to examine the spatiotemporal stability of the song transitions were unavailable.

Alternatively, the zone of admixture could represent secondary contact after the *Z*. *l*. *nuttalli* populations diverged in allopatry. In this case, song divergence would be acting to maintain genetic divergence between the northern and southern populations. Monterey Bay is a site of taxonomic turnover for marine [80] and terrestrial [81–85] organisms. A marine embayment at what is now Monterey Bay is thought to have persisted approximately from 18 million years ago until 600,000 years ago; this marine embayment is a commonly posited mechanism for vicariance in terrestrial taxa [82, 84, 85]. *Z*. *l*. *nuttalli* populations, which now occur continuously around Monterey Bay, may have been fragmented during this time. But genetic divergence between *Z*. *l*. *nuttalli* and *Z*. *l*. *pugetensis*—which we found to be deeper than the genetic break within *Z*. *l*. *nuttalli*—likely occurred during the last glacial maximum (20,000 years ago), so both divergences may be more recent than the marine embayment [29]. For some other taxa, genetic breaks at Monterey Bay are also not consistent with the marine embayment period, suggesting the presence of other mechanisms for vicariance around the bay [83, 86]. Therefore, we cannot rule out allopatry as the original driver of genetic divergence at Monterey Bay.

In terms of which aspects of song are acting as a reproductive barrier, we did not directly examine whether differences in particular song traits—rather than overall song dissimilarity—are acting as barrier to gene flow at Monterey Bay. White-crowned sparrow females use song traits to recognize potential mates [87], assess male quality [57, 88–91], and differentiate between local and non-local songs [38, 92, 93]. Vocal performance is a function of trill rate and trill bandwidth in white-crowned sparrows; high performance songs have higher trill rates and bandwidth [57, 88–91]. Importantly, large trill bandwidth, not low trill minimum frequency itself, indicates high male vocal performance [91]. The northern and southern population differ in minimum and maximum frequency, average trill note length, and trill minimum frequency, but not any bandwidth measures or trill frequency. While these traits vary with habitat structure [47, 94], they have not been shown to directly influence signal salience. Vocal performance does not appear to differ between populations, but females may be using population-level song trait differences to discriminate against non-local songs.

Our results generally support the hypothesis that sexual selection based on the degree of song divergence is reducing gene flow between the northern and southern *Z*. *l*. *nuttalli* populations. We posit that song divergence may have driven this genetic divergence, but with these data, we cannot rule out paleoecological changes that could have resulted in a genetic break at Monterey Bay. Regardless, culturally inherited song divergence appears to be maintaining the genetic break, laying the groundwork for speciation by sexual selection within a subspecies. This study and other recent studies support the ability of cultural dissimilarity to reduce gene flow between populations through assortative mating [77, 95–97], highlighting the influence of gene-culture coevolution on biological evolution.

## Supporting information

S1 Appendix Accession information for vouchered specimens at the Museum of Natural Science at Louisiana State University. (PDF)

S2 Appendix LMM results for song playback experiment. Output and model fit for linear mixed models run on playback experiment data.
(PDF)

S1 Fig Song playback experiment sites. (TIF)

S2 Fig Averaged k = 2 fastSTRUCTURE results for *Z*. *l*. *nuttalli* and *Z*. *l*. *pugetensis*. Populations are sorted from north to south. Pink indicates *Z*. *l*. *pugetensis*, and blue indicates *Z*. *l*. *nuttalli*.
(TIF)

S3 Fig Scatterplot of first two linear discriminants from DAPC. (TIF)

S4 Fig Heat map and boxplot of population-level pairwise Fst values. Populations are sorted north (top right) to south (bottom left). Pink heat map cells represent null values (comparison to self).
(TIF)

S5 Fig PCA of song trait values. (A) The first and second principal components of song trait variation. (B) The first and fourth principal components of song trait variation. (C) Table with variable loadings for statistically significant PCs.
(TIF)

S6 Fig PC1 and PC2 of playback experiment behavioral variables. (TIF)

S7 Fig MDS of songs used to calculate acoustic dissimilarity. (TIF)

S8 Fig Aggression scores for males tested in the playback experiment. Each box plot shows the focal male aggression scores in response to the song stimulus treatment.
(TIF)
