# Supplementary material for: Song and genetic divergence within a subspecies of white-crowned sparrow (Zonotrichia leucophrys nuttalli)
Source: PLoS One. 2024 May 29;19(5):e0304348. doi: 10.1371/journal.pone.0304348 (PMC11135742; doi:10.1371/journal.pone.0304348)
Supplement: S1 Appendix — (PDF) [file pone.0304348.s001.pdf]

Accession Number	Genus	Species	Subspecies	Collection Locality
B25070	Zonotrichia	atricapilla	NA	CA, Riverside Co, San Bernadino Mtns, E. branch Malliard Cyn, 4km SW Kiching Peak
B25071	Zonotrichia	atricapilla	NA	CA, Riverside Co, San Bernadino Mtns, E. branch Malliard Cyn, 4km SW Kiching Peak
B40807	Zonotrichia	atricapilla	NA	WA, Grays Harbor Co, Aberdeen
B45483	Zonotrichia	leucophrys	oriantha	TX, Jeff Davis Co, Miller Ranch, farmhouse 17km W, 2km N Valentine
B48027	Zonotrichia	leucophrys	gambeli	TX, Presidio Co, Miller Ranch, Sierra Vieja, ZH Canyon, area in vicinity of old fort, 15km W, 4.5 km S Valentine
B48038	Zonotrichia	leucophrys	gambeli	TX, Presidio Co, Miller Ranch, Sierra Vieja, ZH Canyon, area in vicinity of old fort, 15km W, 4.5 km S Valentine
B48041	Zonotrichia	leucophrys	gambeli	TX, Presidio Co, Miller Ranch, Sierra Vieja, ZH Canyon, area in vicinity of old fort, 15km W, 4.5 km S Valentine
B53435	Zonotrichia	atricapilla	NA	WA, Skagit Co, Mt Vernon, Franciss Rd
B54875	Zonotrichia	leucophrys	gambeli	TX: Presidio Co, Big Bend Ranch State Park, ca 2 mi. ENE Saucedo Ranch
B62716	Zonotrichia	leucophrys	oriantha	Idaho: Bear Lake Co, Wasatch Range, Paris Flat, 8 miles W Paris, 42 degrees 13' 57.5" N 111 degrees 33' 26.5"
B62729	Zonotrichia	leucophrys	oriantha	Idaho: Bonneville Co, Cambov Range, McCoy Creek, 0.4 miles E Anderson Gulch Rd, 43 degrees 8'29.3"N, 111 degrees 16'14.3"W
B62730	Zonotrichia	leucophrys	oriantha	Idaho: Bonneville Co, Cambov Range, McCoy Creek, 0.4 miles E Anderson Gulch Rd, 43 degrees 8'29.3"N, 111 degrees 16'14.3"W
B73630	Zonotrichia	leucophrys	nuttalli	Commonweal
B73631	Zonotrichia	leucophrys	nuttalli	Commonweal
B73632	Zonotrichia	leucophrys	nuttalli	Commonweal
B73633	Zonotrichia	leucophrys	nuttalli	Commonweal
B73634	Zonotrichia	leucophrys	nuttalli	Abbotts Lagoon
B73635	Zonotrichia	leucophrys	nuttalli	Abbotts Lagoon

B73636	Zonotrichia	leucophrys	nuttalli	Abbotts Lagoon
B73637	Zonotrichia	leucophrys	nuttalli	Abbotts Lagoon
B73638	Zonotrichia	leucophrys	nuttalli	Abbotts Lagoon
B73640	Zonotrichia	leucophrys	nuttalli	Sonoma
B73641	Zonotrichia	leucophrys	hybrid	Sonoma
B73642	Zonotrichia	leucophrys	hybrid	Sonoma
B73643	Zonotrichia	leucophrys	hybrid	Sonoma
B73644	Zonotrichia	leucophrys	hybrid	Sonoma
B73645	Zonotrichia	leucophrys	hybrid	Sonoma
B73646	Zonotrichia	leucophrys	hybrid	Sonoma
B73647	Zonotrichia	leucophrys	hybrid	Sonoma
B73648	Zonotrichia	leucophrys	hybrid	Sonoma
B73649	Zonotrichia	leucophrys	nuttalli	Vallejo
B73650	Zonotrichia	leucophrys	hybrid	Manchester
B73651	Zonotrichia	leucophrys	hybrid	Manchester
B73652	Zonotrichia	leucophrys	hybrid	Manchester
B73653	Zonotrichia	leucophrys	hybrid	Manchester
B73654	Zonotrichia	leucophrys	hybrid	Manchester
B73655	Zonotrichia	leucophrys	hybrid	Manchester
B73656	Zonotrichia	leucophrys	hybrid	Manchester
B73657	Zonotrichia	leucophrys	hybrid	Manchester
B73658	Zonotrichia	leucophrys	hybrid	Manchester
B73659	Zonotrichia	leucophrys	hybrid	Manchester
B73660	Zonotrichia	leucophrys	hybrid	Sinkyone Wilderness State Park
B73661	Zonotrichia	leucophrys	hybrid	Sinkyone Wilderness State Park
B73662	Zonotrichia	leucophrys	hybrid	Sinkyone Wilderness State Park
B73663	Zonotrichia	leucophrys	hybrid	Ferndale

B73664	Zonotrichia	leucophrys	hybrid	Ferndale
B73665	Zonotrichia	leucophrys	hybrid	Ferndale
B73666	Zonotrichia	leucophrys	hybrid	Ferndale
B73667	Zonotrichia	leucophrys	hybrid	Ferndale
B73668	Zonotrichia	leucophrys	hybrid	Ferndale
B73669	Zonotrichia	leucophrys	hybrid	Ferndale
B73670	Zonotrichia	leucophrys	hybrid	Ferndale
B73671	Zonotrichia	leucophrys	hybrid	Ferndale
B73672	Zonotrichia	leucophrys	hybrid	Trinidad
B73673	Zonotrichia	leucophrys	hybrid	Trinidad
B73674	Zonotrichia	leucophrys	hybrid	Trinidad
B73675	Zonotrichia	leucophrys	hybrid	Trinidad
B73676	Zonotrichia	leucophrys	hybrid	Trinidad
B73677	Zonotrichia	leucophrys	hybrid	Trinidad
B73678	Zonotrichia	leucophrys	hybrid	Trinidad
B73679	Zonotrichia	leucophrys	hybrid	Trinidad
B73680	Zonotrichia	leucophrys	hybrid	Trinidad
B73681	Zonotrichia	leucophrys	hybrid	Trinidad
