# Supplementary material for: Song and genetic divergence within a subspecies of white-crowned sparrow (Zonotrichia leucophrys nuttalli)
Source: PLoS One. 2024 May 29;19(5):e0304348. doi: 10.1371/journal.pone.0304348 (PMC11135742; doi:10.1371/journal.pone.0304348)

Song dissimilarity vs. Fst and geographic distance

```
Linear mixed model fit by REML. t-tests use Satterthwaite's method ['lmerModLmerTest']
Formula: song_dissimilarity ~ distance + fst + (1 | male) + (1 | site/treatment_locale)
   Data: no_fst_dataset

REML criterion at convergence: 285.3

Scaled residuals:
     Min       1Q   Median       3Q      Max
-2.07898 -0.72181  0.01475  0.61272  2.58107

Random effects:
 Groups                Name        Variance Std.Dev.
 male                  (Intercept) 0.05512  0.2348
 treatment_locale:site (Intercept) 0.11123  0.3335
 site                  (Intercept) 0.08370  0.2893
 Residual                          0.60415  0.7773
Number of obs: 100, groups:  male, 51; treatment_locale:site, 44; site, 7

Fixed effects:
              Estimate Std. Error        df t value Pr(>|t|)
(Intercept)  1.679e-01  1.951e-01  1.130e+01   0.861    0.407
distance     1.322e-07  8.041e-08  8.175e+01   1.644    0.104
fst         -5.602e+00  4.097e+00  2.279e+01  -1.367    0.185

Correlation of Fixed Effects:
         (Intr) distnc
distance  0.028
fst      -0.587 -0.246
```

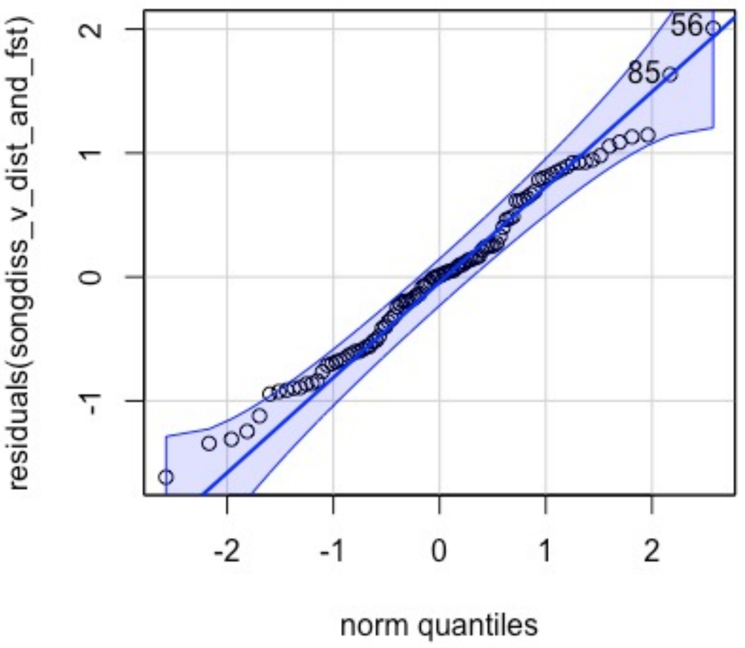

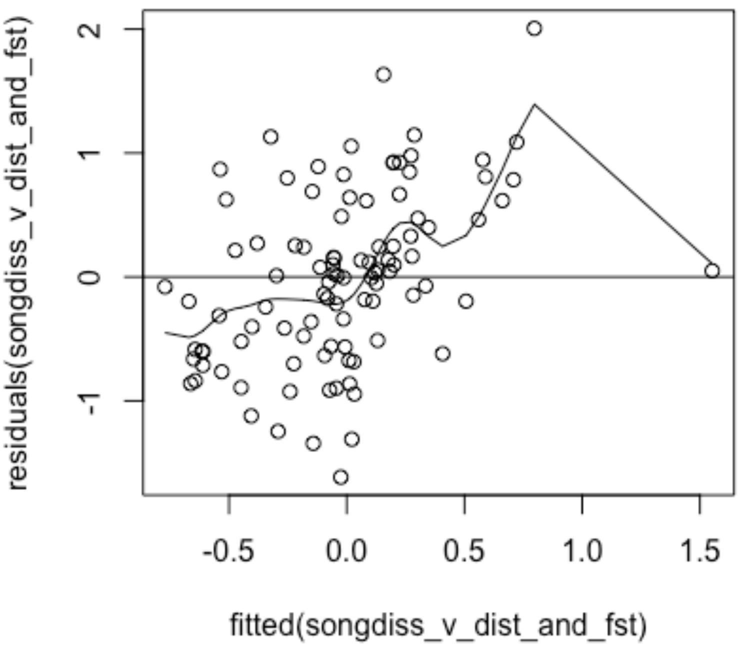

Song discrimination vs. song dissimilarity, reduced dataset (males w/o Fst value removed)

```
Linear mixed model fit by maximum likelihood . t-tests use Satterthwaite's method ['lmerModLmerTest']
Formula: pc1_diff ~ song_dissimilarity + (1 | male)
   Data: remove_singles

     AIC      BIC   logLik deviance df.resid
   306.5    317.0   -149.3    298.5       98

Scaled residuals:
     Min       1Q   Median       3Q      Max
-1.60169 -0.45810 -0.07637  0.47448  2.06223

Random effects:
 Groups   Name        Variance Std.Dev.
 male     (Intercept) 0.5816   0.7626
 Residual             0.6567   0.8103
Number of obs: 102, groups:  male, 51

Fixed effects:
                   Estimate Std. Error      df t value Pr(>|t|)
(Intercept)          0.0783     0.1337 51.0755   0.586   0.5606
song_dissimilarity   0.1823     0.1066 87.4749   1.711   0.0907 .
---
Signif. codes:  0 ‘***’ 0.001 ‘**’ 0.01 ‘*’ 0.05 ‘.’ 0.1 ‘ ’ 1

Correlation of Fixed Effects:
            (Intr)
sng_dssmlrt 0.040
```

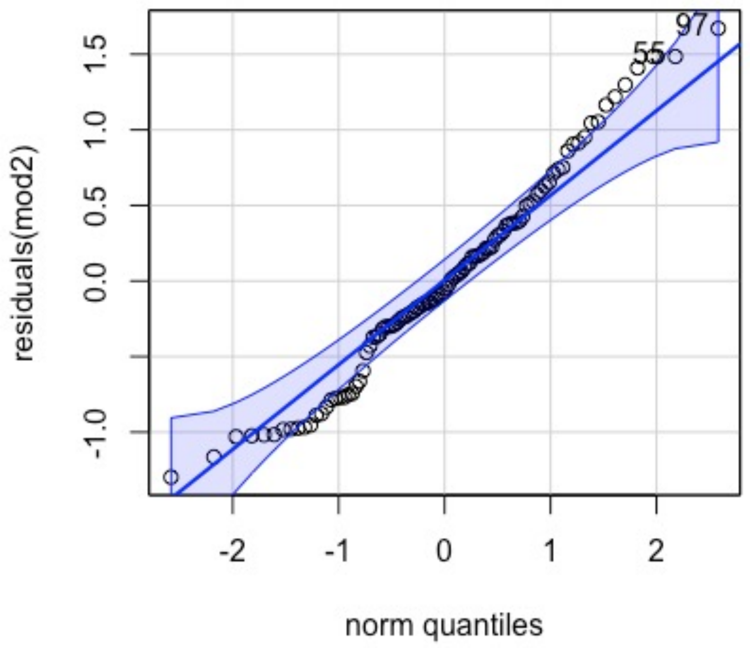

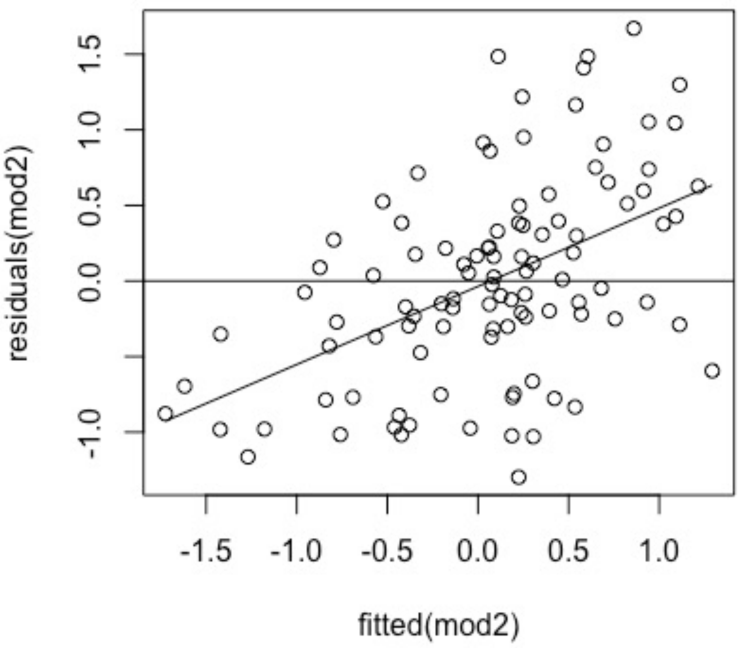

Song discrimination vs. song dissimilarity, Including males with missing Fst values

```
Linear mixed model fit by maximum likelihood . t-tests use Satterthwaite's method ['lmerModLmerTest']
Formula: pc1_diff ~ song_dissimilarity + (1 | male)
   Data: final_dataset2

     AIC      BIC   logLik deviance df.resid
   376.6    387.9   -184.3    368.6      120

Scaled residuals:
     Min       1Q   Median       3Q      Max
-1.67720 -0.43068 -0.06748  0.44197  2.12155

Random effects:
 Groups   Name        Variance Std.Dev.
 male     (Intercept) 0.7637   0.8739
 Residual             0.6119   0.7822
Number of obs: 124, groups:  male, 62

Fixed effects:
                    Estimate Std. Error        df t value Pr(>|t|)
(Intercept)          0.01113    0.13135  61.96632   0.085  0.93272
song_dissimilarity   0.25460    0.09121 103.18980   2.792  0.00625 **
---
Signif. codes:  0 ‘***’ 0.001 ‘**’ 0.01 ‘*’ 0.05 ‘.’ 0.1 ‘ ’ 1

Correlation of Fixed Effects:
            (Intr)
sng_dssmlrt 0.003
```

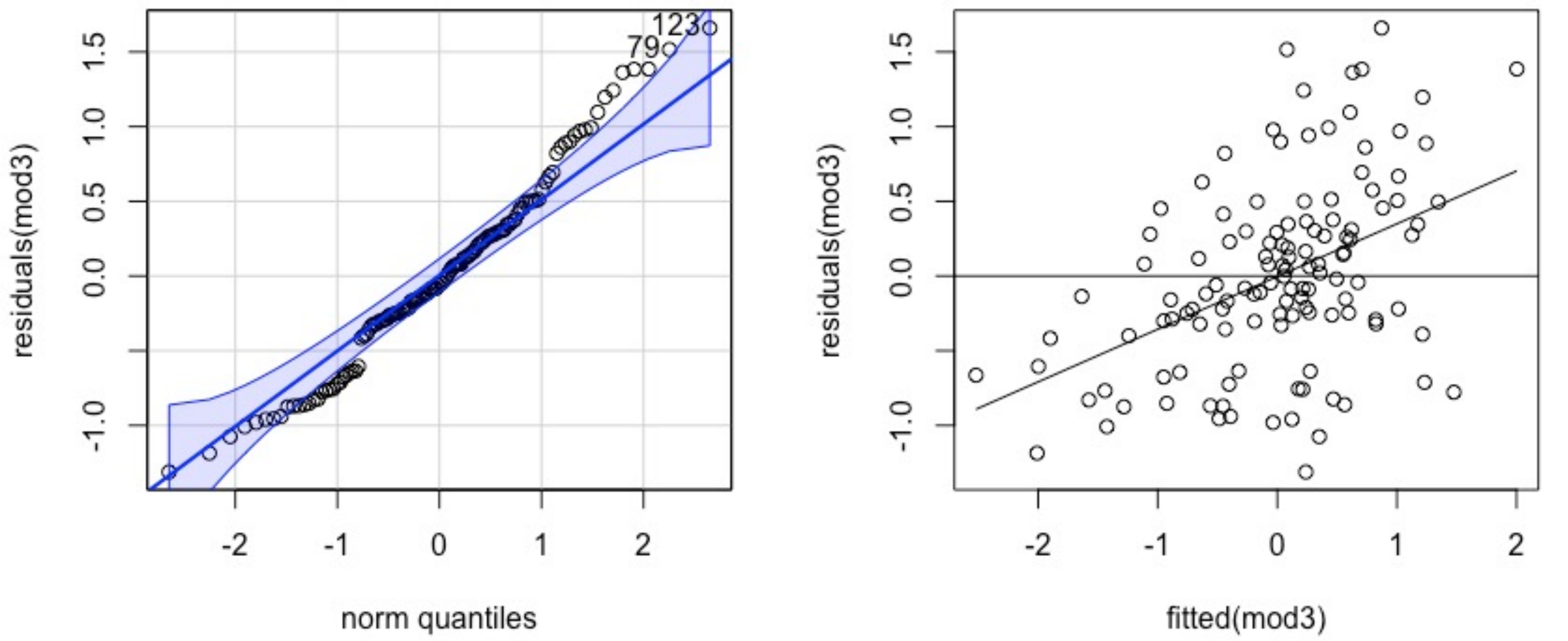

Supplement: S2 Appendix — Output and model fit for linear mixed models run on playback experiment data. (PDF) [file pone.0304348.s002.pdf]
