# Supplementary figures and images for: Song and genetic divergence within a subspecies of white-crowned sparrow (Zonotrichia leucophrys nuttalli)
Source: PLoS One. 2024 May 29;19(5):e0304348. doi: 10.1371/journal.pone.0304348 (PMC11135742; doi:10.1371/journal.pone.0304348)

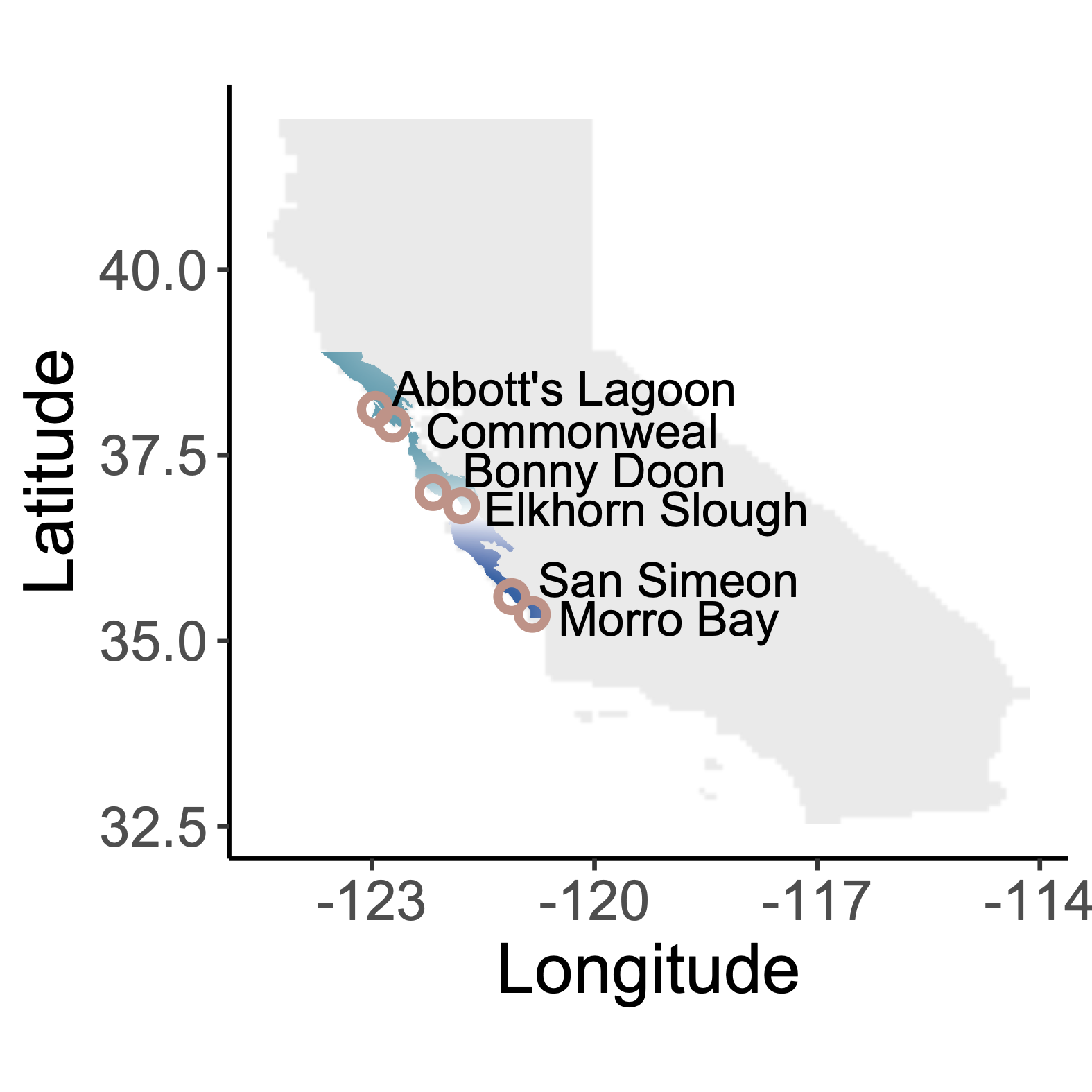

Supplement: S1 Fig — (TIF) [file pone.0304348.s003.tif]

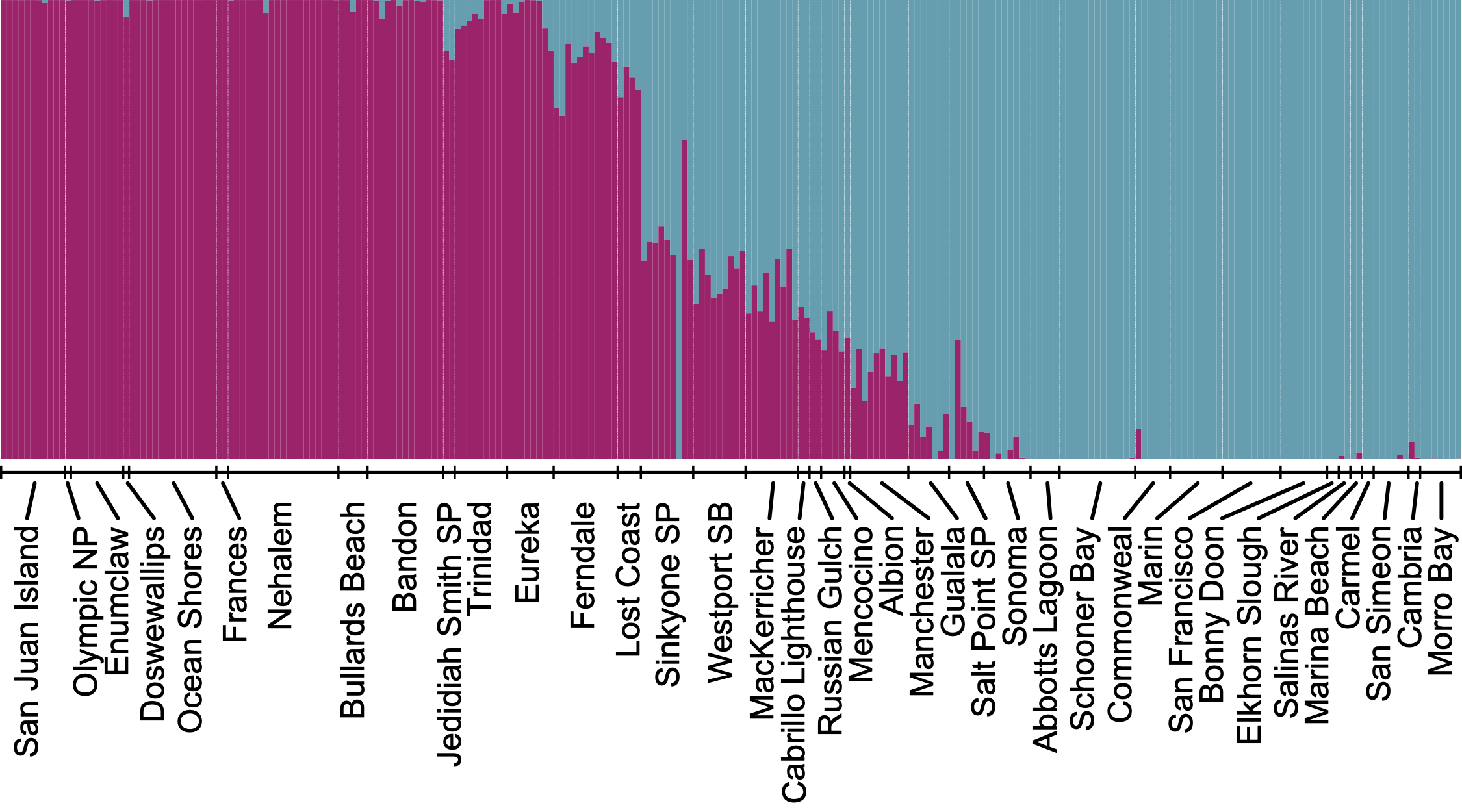

Supplement: S2 Fig — Populations are sorted from north to south. Pink indicates Z. l. pugetensis, and blue indicates Z. l. nuttalli. (TIF) [file pone.0304348.s004.tif]

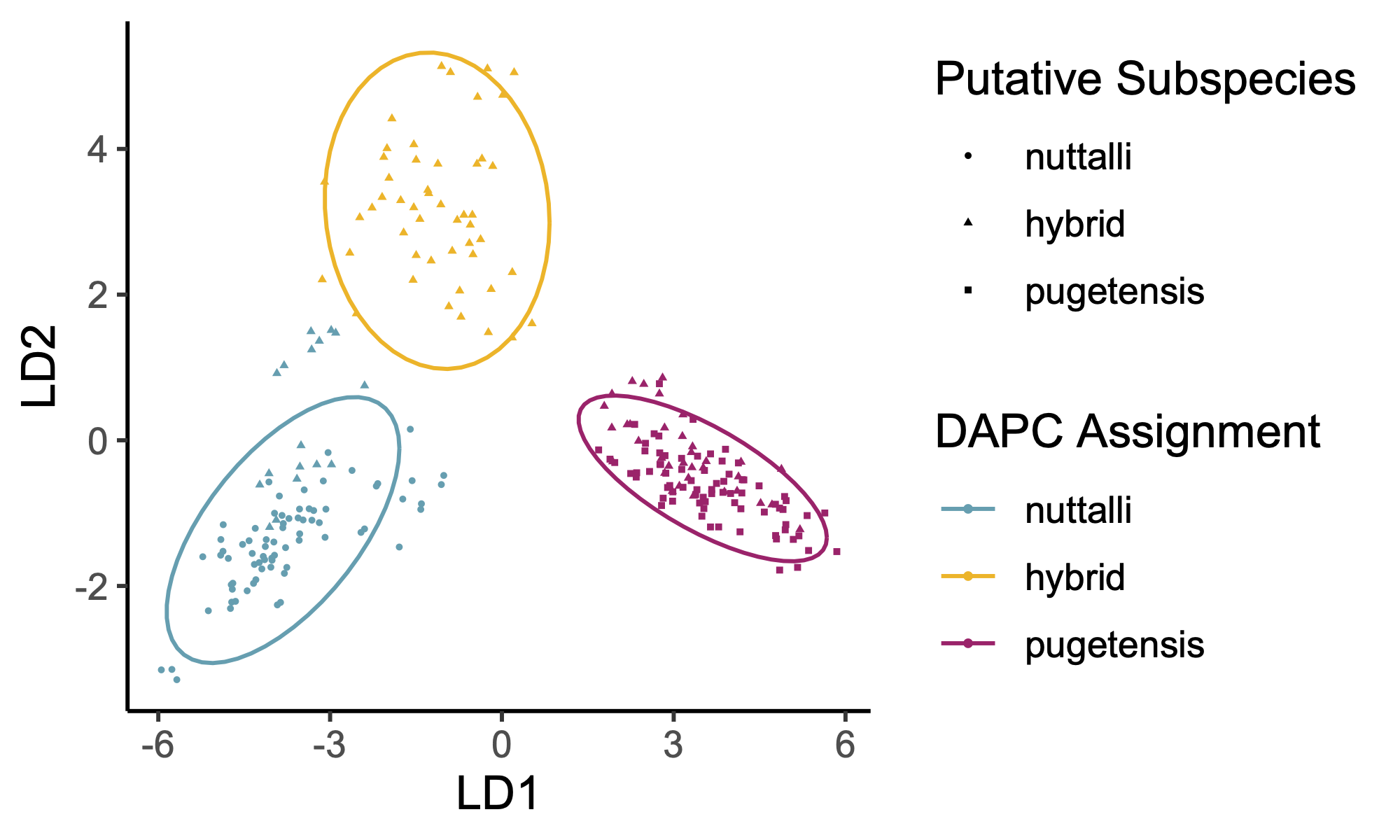

Supplement: S3 Fig — (TIF) [file pone.0304348.s005.tif]

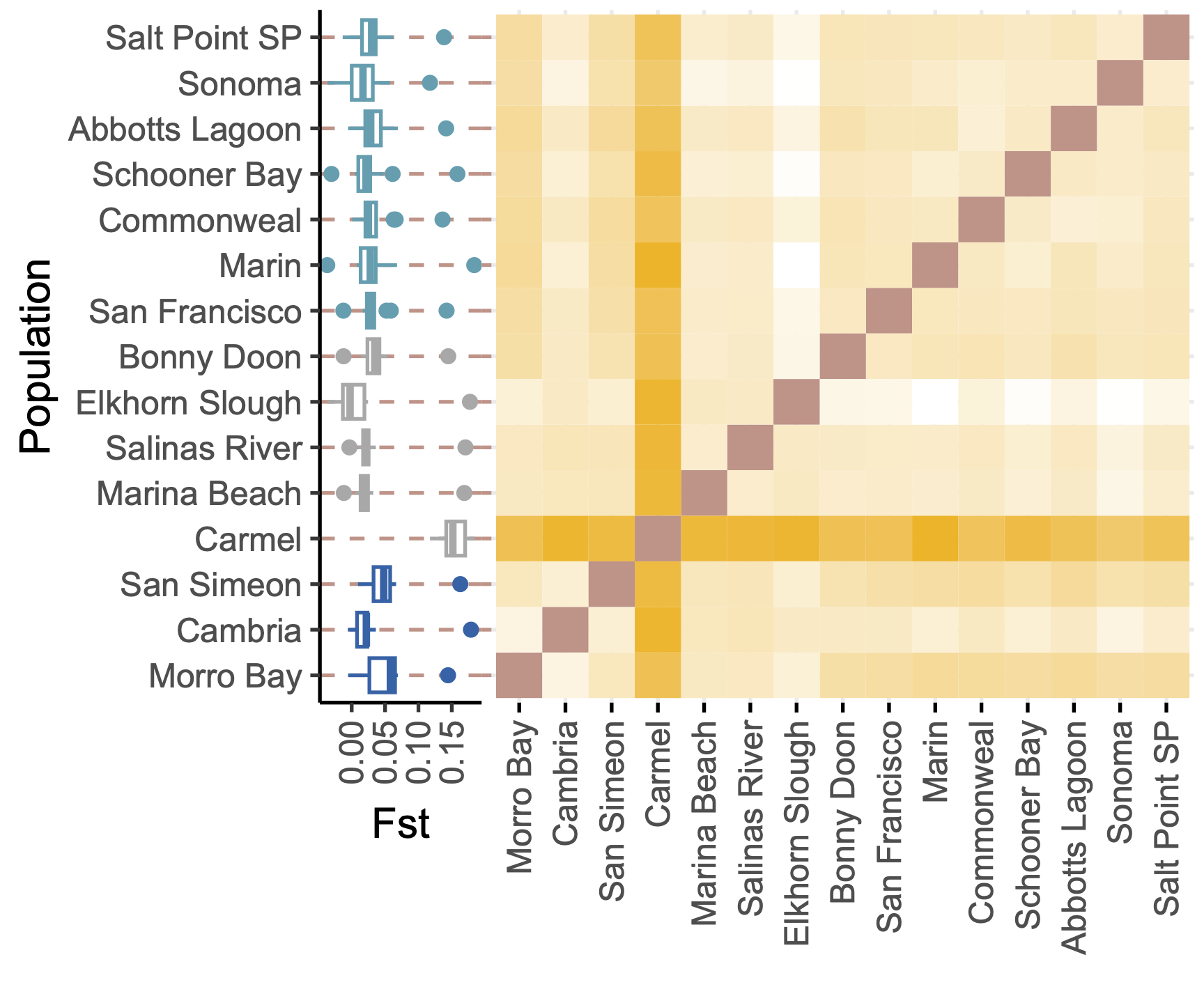

Supplement: S4 Fig — Populations are sorted north (top right) to south (bottom left). Pink heat map cells represent null values (comparison to self). (TIF) [file pone.0304348.s006.tif]

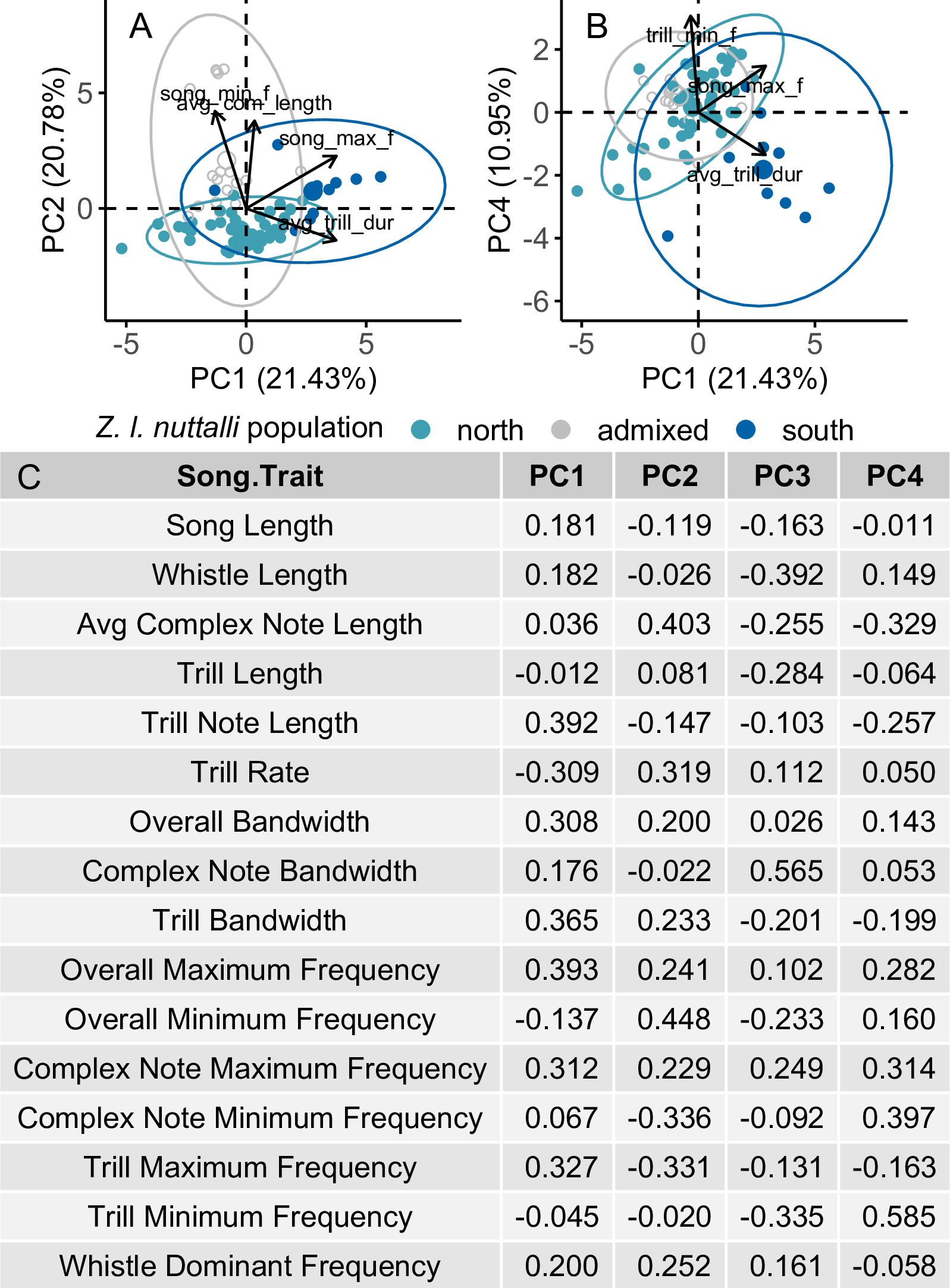

Supplement: S5 Fig — (A) The first and second principal components of song trait variation. (B) The first and fourth principal components of song trait variation. (C) Table with variable loadings for statistically significant PCs. (TIF) [file pone.0304348.s007.tif]

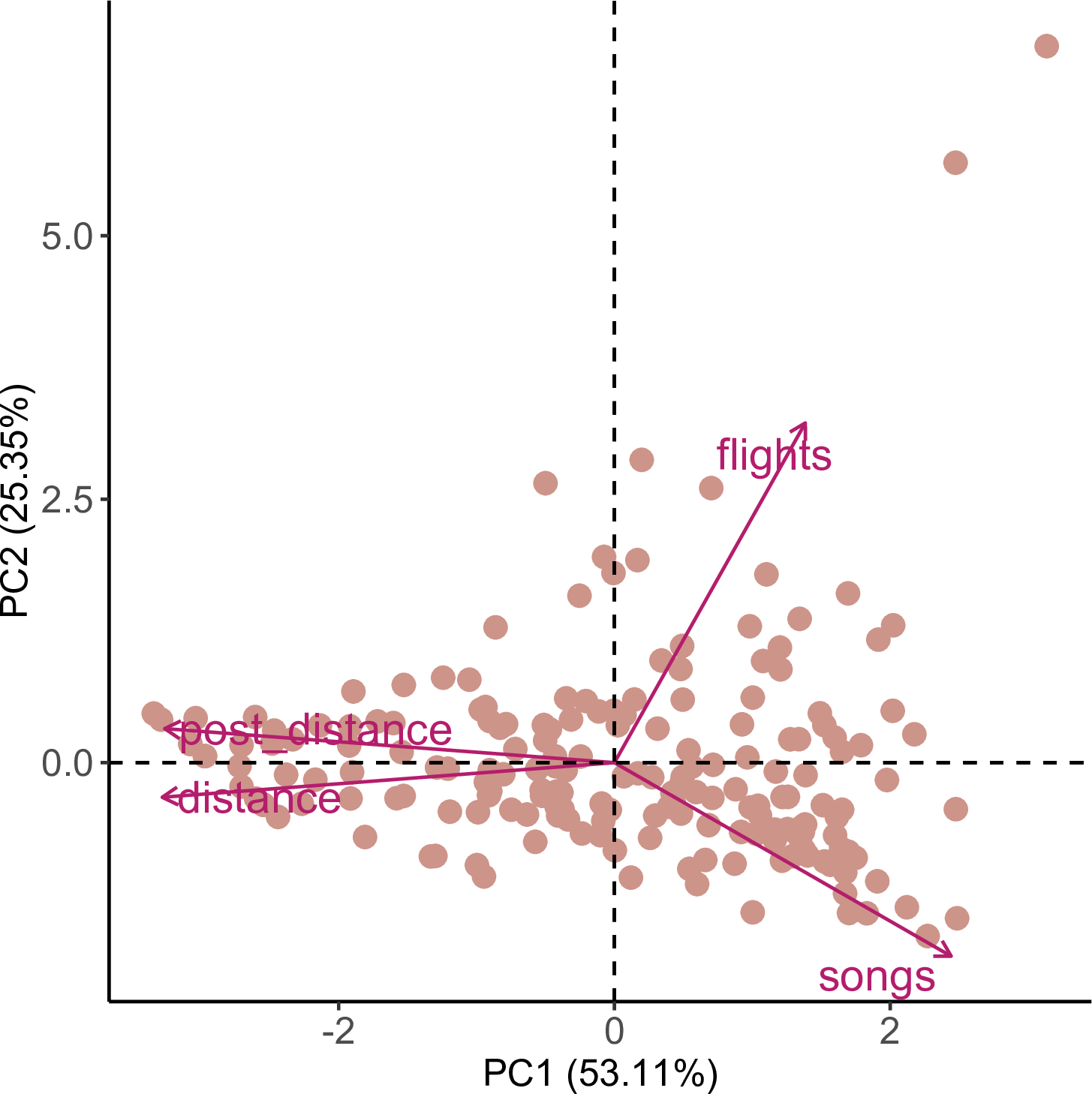

Supplement: S6 Fig — (TIF) [file pone.0304348.s008.tif]

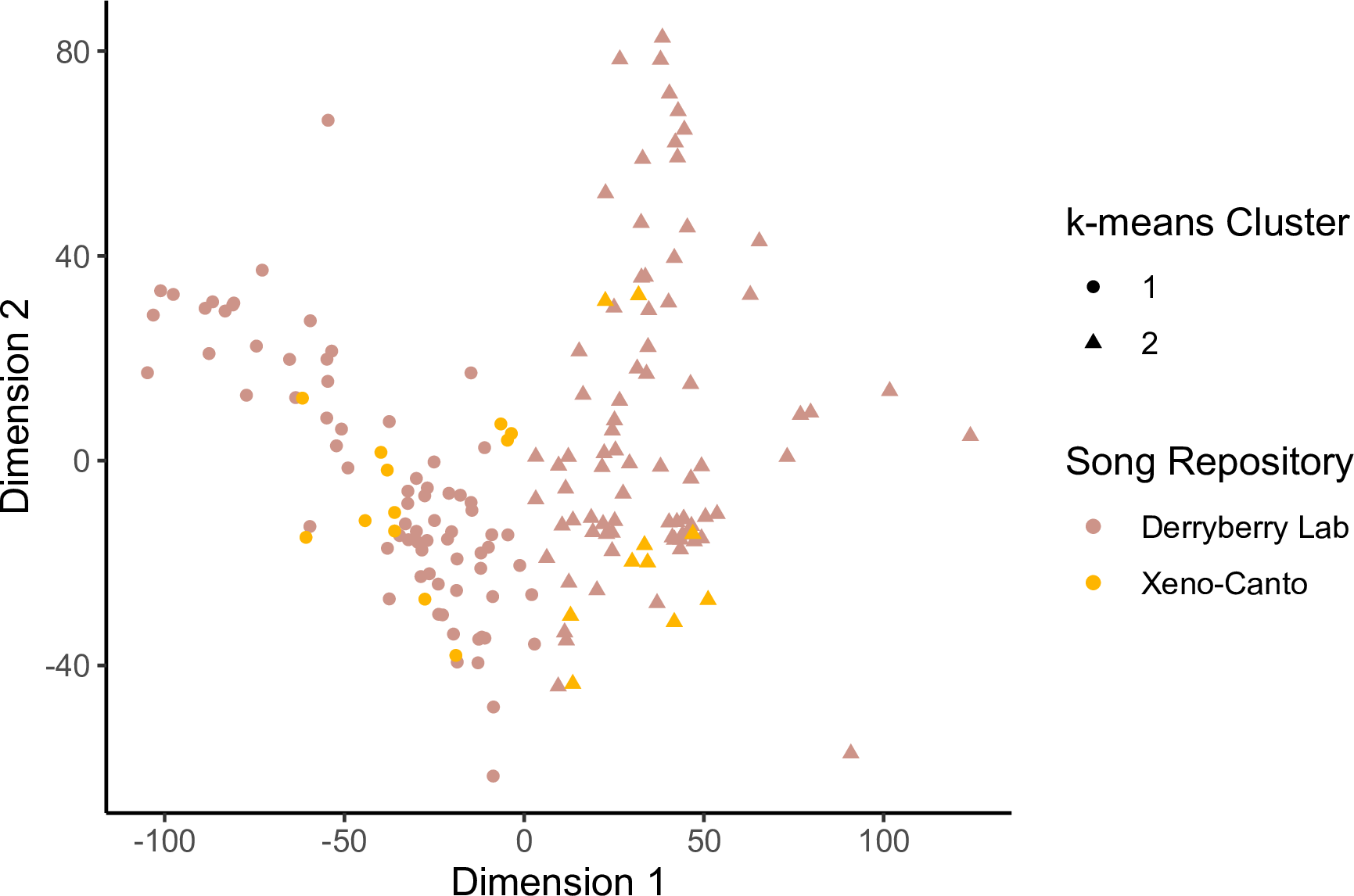

Supplement: S7 Fig — (TIF) [file pone.0304348.s009.tif]

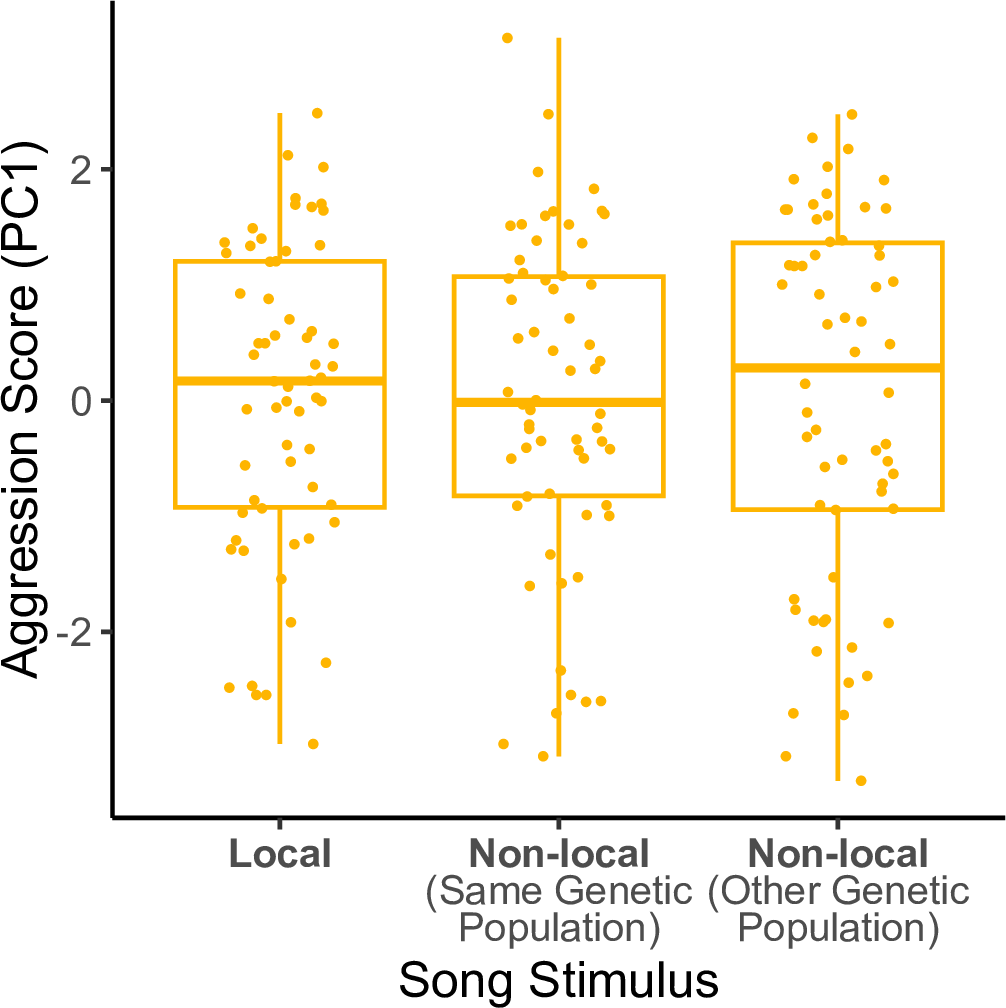

Supplement: S8 Fig — Each box plot shows the focal male aggression scores in response to the song stimulus treatment. (TIF) [file pone.0304348.s010.tif]
